# A new flexible model for maintenance and feeding expenses that improves description of individual growth in insects

**DOI:** 10.1038/s41598-023-43743-1

**Published:** 2023-10-05

**Authors:** Karl Mauritsson, Tomas Jonsson

**Affiliations:** 1 Ecological Modelling Group, School of Bioscience, University of Skövde, Skövde, Sweden; 2 Ecological and Environmental Modeling, Department of Physics, Chemistry and Biology, Linköping University, Linköping, Sweden

**Keywords:** Theoretical ecology, Entomology, Animal physiology, Ecological modelling

## Abstract

Metabolic theories in ecology interpret ecological patterns at different levels through the lens of metabolism, typically applying allometric scaling to describe energy use. This requires a sound theory for individual metabolism. Common mechanistic growth models, such as ‘von Bertalanffy’, ‘dynamic energy budgets’ and the ‘ontogenetic growth model’ lack some potentially important aspects, especially regarding regulation of somatic maintenance. We develop a model for ontogenetic growth of animals, applicable to ad libitum and food limited conditions, based on an energy balance that expresses growth as the net result of assimilation and metabolic costs for maintenance, feeding and food processing. The most important contribution is the division of maintenance into a ‘non-negotiable’ and a ‘negotiable’ part, potentially resulting in hyperallometric scaling of maintenance and downregulated maintenance under food restriction. The model can also account for effects of body composition and type of growth at the cellular level. Common mechanistic growth models often fail to fully capture growth of insects. However, our model was able to capture empirical growth patterns observed in house crickets.

## Introduction

Animals can be considered as regulation processes that acquire energy from the environment and convert it into more ordered forms of biomass in order to grow, produce offspring and maintain homeostasis. Energy conversion occurs through the processes of metabolism under a high level of coordination, feedback and integration^1^. Variation in the rate of metabolism among animals has important ecological implications through its effects on, for example, abundance, distribution, colonizing success and global variation in species richness^2^. Metabolic theories in ecology interpret processes at different ecological levels through the lens of metabolism. Much work has been published within the framework known as the *metabolic theory of ecology* (MTE)^3^, which is based on allometric scaling and temperature dependence of metabolic rate, *R*, for individual organisms:

1 $$R = \alpha W^{\beta } e^{ - \varepsilon /(\kappa T)}$$

Here *W* is body mass, *T* absolute temperature, *α* a normalization constant, *β* an allometric exponent, *ε* activation energy and *κ* is Boltzmann’s constant^4,5^. Traditionally, metabolic rate has often focused on the *resting metabolic rate* (the metabolic rate of a non-feeding inactive organism) and this is normally what is measured empirically. Of interest in metabolic ecology is usually the *field* (or active) *metabolic rate* instead (the *total metabolic rate* of a free-living organism, spending energy on foraging and other activities). This is usually considerably higher, but often considered proportional to the resting metabolic rate (and thus allometrically related to body size as well^3^).

Comparisons of species from various taxa has been used to argue that the interspecific allometric exponent (across species of varying adult body size) is often close to 3/4^6,7^. Thus, MTE in its original form applies *β* = 3/4 and assumes that individual metabolic rate sets the rates for many other biological activities, which generates patterns for various ecological processes at higher levels, such as population growth rates, trophic interactions and biomass production^3^. This view has been criticized for missing that metabolism often responds to biological processes rather than drives them, ignoring the importance of regulation processes^1^. Furthermore, studies indicate that there is no universal value of the allometric exponent; interspecific relationships of adults^8–12^ has yielded values that vary from slightly less than 0.5 to slightly more than 1 for different taxa^13^. Within species, the exponent may also change during the lifetime of an individual^14^. In addition, non-linear scaling of metabolic rates has been reported^13,15–17^.

Many models have been proposed to explain observed apparent allometric scaling of metabolic rate, including views where metabolic rates are limited by internal resource distribution networks^18,19^, fluxes across exchange surfaces^20,21^, composition of body components with different metabolic activity^22,23^ and resource demand of different whole-body processes^24,25^. For example, Shestopaloff^19^ argued that metabolic rate is limited by costs of transportation across cells, implying that variation in cell size explains differences in allometric exponents between organisms that grow by cell enlargement, cell division or a combination of both. As a consequence of all this, metabolism is now often seen as a result of several interactive and overlapping processes with different rates in different tissues at different phases during ontogeny^26^. A sound metabolic theory requires a growth model that can capture such aspects.

In order to give a mechanistic description of individual growth, we here build on some of the previously discussed metabolic approaches by describing total metabolic rate as the sum of several components, accounting for differences in maintenance and growth costs due to life stage, tissue composition and cell growth type.

To be really useful as a foundation for metabolic theory, a growth model must be able to deal with resource limitation and its effect(s) on metabolism and growth. Under food restriction, resting metabolic rates are generally lowered^27^. A trade-off between fast growth and maintenance affect this pattern^28^ and may include downregulation of ‘non-necessary’ maintenance processes^29–33^, such as maintenance of the immune system^34^. However, many mechanistic growth models that consider food limitation^35–37^ fail to consider effects of metabolic regulation on maintenance (see Note SI1.2).

Furthermore, common mechanistic growth models applied to ad libitum conditions^38–40^ often fail to describe growth of insects, overpredicting growth rate at early ontogeny, underpredicting it at later stages and failing to accurately predict terminated growth^41^. A growth model that considers relevant aspects of metabolic regulation and growth may however do this. Such a model should be able to capture the following potentially relevant aspects: (1) non-linear allometric scaling of metabolic components; (2) maintenance regulation under food restriction; (3) costs of finding and processing food; (4) effects of body composition on costs for growth and maintenance; (5) differences in costs for growth and maintenance between somatic and reproductive tissue; (6) effects of proportions of cell growth and cell division on costs for growth and maintenance. In order to improve the foundation for analysis of ecological patterns at higher levels in terms of metabolism, we propose a flexible model for ontogenetic and post-mature growth, able to account for these aspects. Total metabolism is decomposed into several components, including costs for maintenance, growth, activity and food processing. Growth is expressed as the net result of assimilation and metabolic expenses. We call this model the Maintenance-Growth Model (MGM) to highlight the trade-off between maintenance and growth. MGM is a general framework that includes many aspects, but can be simplified for specific situations depending on the biology of a particular organism. The new model is here presented, derived and demonstrated via numerical simulations and some comparisons with previous data, but detailed empirical model validation is saved for forthcoming work.

## The maintenance-growth model

**Figure 1:**
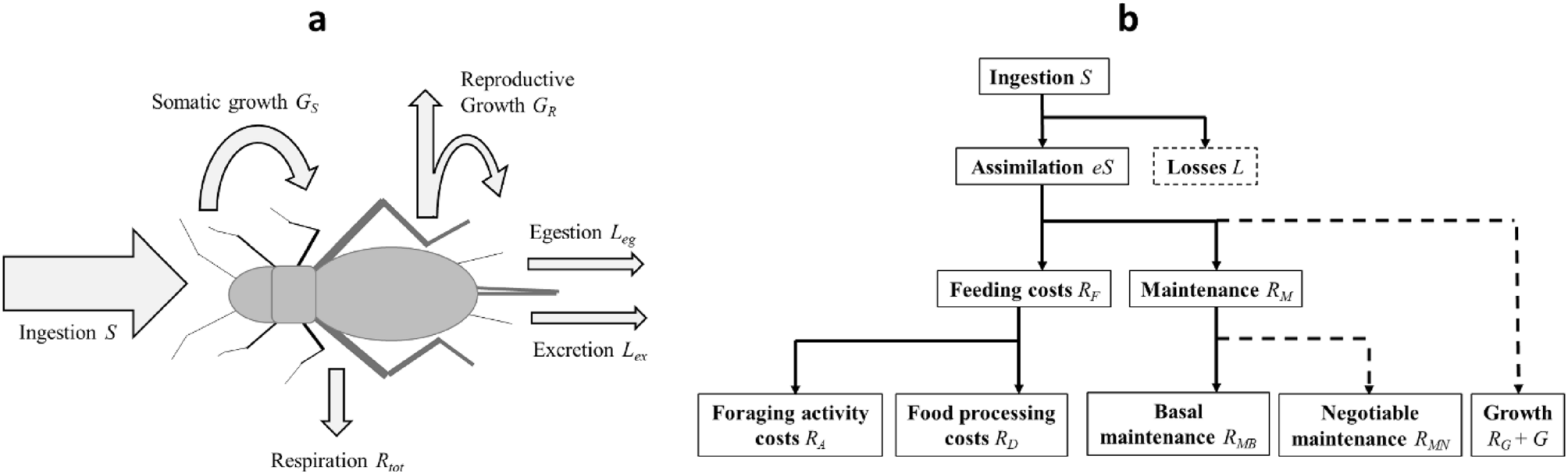
(**a**) Energy balance of a growing animal. Arrows represent fluxes of energy. *Ingestion S* is all energy that enters the animal through consumed resources. *Respiration R*_*tot*_ represents energy that drives all metabolic processes (Eq. 6), energy that is eventually released as heat to the environment. *Somatic growth G*_*S*_ is energy becoming bounded in synthesized somatic biomass. *Reproductive growth G*_*R*_ is energy becoming bounded in biomass of gonads and produced sperms/eggs/offspring. The transformation of energy from ingested food into usable forms include some energetic losses. *Egestion L*_*eg*_ is losses through faeces, whereas *excretion L*_*ex*_ is losses through urine and other excretes. (**b**) Energy flow and allocation according to MGM. Boxes represent available energies or energetic costs. Solid arrows represent prioritized metabolic processes at each ‘energetic level’ whose costs are paid first, while dashed arrows represent processes that can be down-regulated in response to what is available at each level after prioritized costs have been paid.

The basic features of MGM for ontogenetic and post-mature growth of a non-reproducing animal is here derived, based on the energy balance between ingestion (*S*), metabolic expenses (*R*_*tot*_), growth (*G*) and losses (*L*) (Fig. 1a). Derivation and specification of individual model components can be found in “Methods” (“Derivation and specification of model components”).

The basic energy balance is expressed as:

2 $$S = R_{tot} + G + L$$

The ingestion rate *S* is ingested energy per unit of time *t* and is assumed to be a function of food availability (*φ*) and body mass (*W*) (see “Ingestion rate”):

3 $$S = S(\varphi ,W)$$

Total growth (*G* = *G*_*S*_ + *G*_*R*_) includes both somatic and reproductive growth, whereas total loss (*L* = *L*_*eg*_ + *L*_*ex*_) includes losses through egestion and excretion. A fraction (1 − *e*) of the ingested energy is lost through egestion and excretion, where 0 < *e* < 1 is the assimilation efficiency, mainly related to food type and quality. The energy balance can thus be expressed as a balance between assimilation rate (*eS*), total metabolic rate (*R*_*tot*_) and growth (*G*):

4 $$eS = R_{tot} + G$$

The rate of increase of energy bounded in synthesized biomass (*G*) is proportional to the growth rate (*dW*/*dt*):

5 $$G = E_{M} (W) \cdot \frac{dW}{{dt}}$$

Here *E*_*M*_(*W*) is the average mass-specific energy content in synthesized body tissue at body mass *W* (which depends on the relative proportions of carbohydrates, proteins and lipids and, as such, may be affected by the proportion of somatic to reproductive tissue, see “Energy content of synthesized body tissue”).

The total metabolic rate is divided into maintenance cost (*R*_*M*_), feeding cost (*R*_*F*_) and growth overhead cost (*R*_*G*_):

6 $$R_{tot} = R_{M} + R_{F} + R_{G}$$

Growth overhead cost (*R*_*G*_) includes all metabolic costs of growth, such as overhead costs for assembling macromolecules from monomers, and is proportional to the growth rate:

7 $$R_{G} = E_{S} (W) \cdot \frac{dW}{{dt}}$$

Here *E*_*S*_(*W*) is the specific growth overhead cost at body mass *W* (which may be different for somatic and reproductive tissue as well as for somatic cell division and somatic cell growth, see “Growth overhead costs”).

Feeding cost (*R*_*F*_) includes all metabolic costs for searching and processing food and is assumed to be a monotonically increasing function of *S* (see “Feeding costs”):

8 $$R_{F} = R_{F} (S),\quad \quad \frac{{dR_{F} }}{dS} > 0$$

Maintenance *R*_*M*_ is energy spent on processes that keep the animal alive and in good shape, including maintenance of ion potentials across membranes, cell repair, immune activities and thermoregulation (in endotherms). It is assumed that maintenance costs are affected by food availability (*φ*), body mass (*W*) and body composition (a function of *W*) in a way that is dependent on the life history strategy of the animal (see “Maintenance costs”):

9 $$R_{M} = R_{M} (\varphi ,W)$$

Altogether, the relations above define how energy is acquired and utilized (Fig. 1b) and they can be combined and rearranged into a general growth equation for ontogenetic and post-mature growth under ad libitum as well as food limited conditions, describing how the growth rate *dW*/*dt* depends on assimilation rate (*eS*), maintenance costs (*R*_*M*_) and feeding costs (*R*_*F*_), including a life history determined trade-off between maintenance costs and growth-related costs (*R*_*G*_ + *G*):

10 $$\begin{gathered} eS = R_{tot} + G = R_{M} (\varphi ,W) + R_{F} (S) + E_{S} (W) \cdot \frac{dW}{{dt}} + E_{M} (W) \cdot \frac{dW}{{dt}} \Leftrightarrow \hfill \\ \frac{dW}{{dt}} = \frac{1}{{E_{M} (W) + E_{S} (W)}}\left[ {eS(\varphi ,W) - R_{F} (S) - R_{M} (\varphi ,W)} \right] \hfill \\ \end{gathered}$$

Potential formulations for functions *E*_*M*_(*W*), *E*_*S*_(*W*), *S*(*φ*,*W*), *R*_*F*_(*S*) and *R*_*M*_(*φ*,*W*) are described in the “Methods” (“Derivation and specification of model components”) by Eqs. (13), (14), (12), (18) and (29). Including all of this unavoidably leads to a complex model with many parameters. However, the model can be significantly simplified under certain assumptions (see “Possible model simplifications”).

MGM is similar in some respects to other previously presented growth models (see Supplementary Information, Note SI1) but differ in some important aspects, mainly the flexible level of details and the division of maintenance costs into a ‘non-negotiable’ part (processes necessary to keep the organism alive) and a ‘negotiable’ part (processes that keep the organism in good shape, but that may be downregulated in order to save energy).

## Results

The growth model (MGM) developed here (Eq. 10) includes five basic components (*E*_*M*_, *E*_*S*_, *S*, *R*_*F*_ and *R*_*M*_), each of which can be described in varying degrees of mathematical complexity, depending on the biology of the organism, the process believed to be important and level of details wanted. To demonstrate the behaviour of MGM under every possible scenario is impossible and we choose to focus here on (1) comparing some predictions of MGM under ad libitum conditions and a number of other simplifying assumptions (Eq. 33) to those of a general formulation of ontogenetic growth (Eq. 34), hereafter termed the Generalized Standard Growth Model (GSGM) representing many previous mechanistic growth models (Note SI1.1), and (2) illustrating some behaviour of MGM under food restriction. In this we make use of experimental data from a previous study on house crickets (Jonsson^42^, see “Empirical data from previous study”). In addition, (3) the general behaviour of MGM under five specific scenarios (effects of (i) feeding costs, (ii) body composition, (iii) growth strategy, (iv) allocation to negotiable maintenance and (v) allocation to reproduction) are demonstrated in Note SI2.3–7.

### Ad libitum conditions

**Figure 2:**
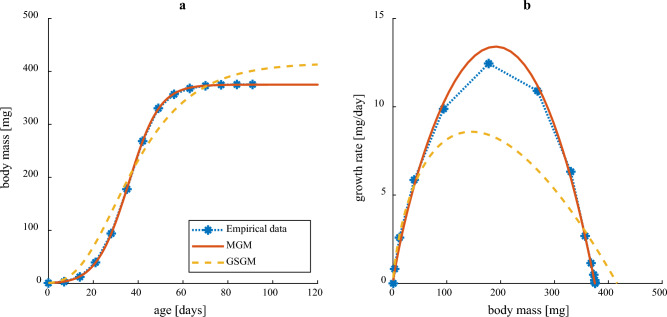
Empirical growth curves for house crickets reared under near ad libitum conditions^42^ and growth curves predicted by the simplest relevant version of MGM (Eq. 33) and GSGM (Eq. 34) with application of optimized parameter values (Table SI3). (**a**) Body mass vs. age. Corresponding goodness of fit measure (Eq. 35) were 0.995 (MGM) and 0.845 (GSGM). (**b**) Growth rate vs. body mass.

The observed average growth of house crickets reared under near ad libitum conditions^42^ was sigmoid (Fig. 2a) and produced a near-symmetrical hump-shaped growth rate curve (Fig. 2b). These trajectories were well captured by MGM, but good agreement was not possible to obtain with GSGM (Fig. 2), using optimized parameters for both models (“Numerical model comparisons”, Table SI3). The inability of GSGM to capture the growth of an insect seen here, is further demonstrated in Note SI2.1. Using two dimensionless key properties of growth trajectories we compared empirical values of these to MGM and GSGM predicted values (Table SI5), and showed that empirical values are impossible to obtain by GSGM. More specifically, the observed ratio between body mass at maximum growth rate and ultimate body mass was found to be considerably larger than the largest value that GSGM is able to predict (Fig. SI2a). Furthermore, it is required that the supply term of the GSGM equation has an unrealistically low value of the allometric exponent to predict the observed ratio between maximum growth rate and average life-time growth rate (Fig. SI2b). Two other growth models from the literature^37,43^, were able to yield quite good agreements with empirical curves, but these models have other deficiencies (see Note SI2.1).

### Food restriction

**Figure 3:**
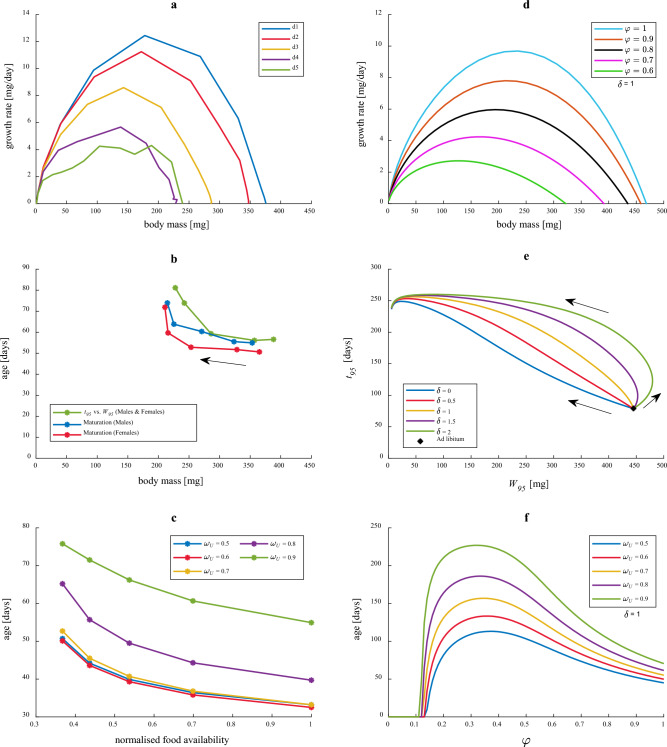
Experimentally observed and MGM-predicted growth behaviour under food restriction. (**a**) Observed growth rate vs. body mass for low (d1) to high density cohorts (d5) of house crickets^42^. All cohorts were exposed to the same total feeding rate, but starting densities varied (d1 = 5, d2 = 10, d3 = 20, d4 = 40, d5 = 80 individuals) and observed mortality increased with starting density. Low-density cohorts (d1) were at or very near ad libitum conditions. (**b**) Observed *t*_95_ vs. *W*_95_ and sex-specific age and body mass at maturation (imago emergence) in food-limited cohorts of house crickets with increased starting density in the direction of the arrow. For the estimation of *W*_95_ and *t*_95_ (age and size when reaching 95% of ultimate body mass) from empirical data^42^, see “Empirical data from previous study”. (**c**) Observed age when specified fractions *ω*_*U*_ of the ultimate body mass is reached for varying levels of food availability. The *normalised food availability* = min[log_10_(*d*_*i*_)]/log_10_(*d*_*i*_) yields a measure between zero and one, based on the initial cohort size *d*_*i*_. (**d**) MGM predictions of growth rate vs. body mass for different fixed levels of relative food acquirement *φ*. (**e**) MGM predictions of *t*_95_ vs. *W*_95_ for varying relative food acquirement* φ* and different values of model parameter *δ*. Each line represents a series of connected data points showing how *t*_95_ and *W*_95_ change with decreasing food availability in the direction of the arrows, for a specified value of *δ*. (**f**) MGM predictions of age when specified fractions *ω*_*U*_ of the ultimate body mass is reached for varying relative food acquirement* φ*.

Growth rates of food restricted cohorts of house crickets were considerably reduced relative to ad libitum (low density cohorts), and more so in cohorts with greater density of individuals where competition for limited resources was larger (Fig. 3a). The end results were reduced size and increased age at (i) maturation and (ii) when reaching 95% of maximum experimentally observed size (Fig. 3b). The ages when specified fractions of the ultimate body mass were reached increased with decreased food availability (Fig. 3c). Many of the experimentally observed patterns can be qualitatively reproduced by MGM. More specifically, simulations of Eq. (10) (as specified by Eq. (SI8) with parameter values from Table SI2) show how increasing food restriction affects MGM growth trajectories in a qualitatively similar way as observed experimentally. That is, growth rates are decreased with reduced food availability (Fig. 3d), measured as relative food acquirement *φ* (ratio of realised ingestion rate to ad libitum ingestion rate at current body size). Furthermore, size (*W*_95_) and age (*t*_95_) when reaching 95% of ultimate size predicted by MGM (Fig. 3e) follow patterns of decreased size and increased age with increased food restriction, as qualitatively observed in experiments (Fig. 3b). However, the exact effect is highly dependent on how fast negotiable maintenance costs are downregulated with decreased relative food acquirement *φ*, as measured by the parameter *δ* (Eqs. (24)–(25)). For *δ* ≲ 1, MGM predicts monotonically decreasing *W*_95_ and increasing *t*_95_ with increasing food restriction. MGM predicted ages when specified fractions of the ultimate body mass are reached increase with decreased food availability (Fig. 3f), the same qualitative pattern as experimentally observed (Fig. 3c). However, qualitatively different predictions of MGM can be obtained by changing model parameters (see Fig. SI4).

### Additional effects

More detailed effects of food restriction in MGM are demonstrated in Note SI2.2, showing reductions in maintenance, growth rate, ultimate body size and metabolic rates at rest and activity (Figs. SI3-4) for some “realistic” parameter values (Table SI2). Effects of increased feeding costs (costs for foraging and food processing) are illustrated in Note SI2.3, showing reduced growth rate and ultimate size (Fig. SI5). MGM-predicted effects of chemical body composition and growth strategy at the cellular level are described in Note SI2.4–5, showing altered growth rates (Figs. SI6-8). Effects of allocation to negotiable maintenance are demonstrated in Note SI2.6, showing altered growth and allometric scaling of metabolic rates (Fig. SI9), and effects on *W*_95_ and *t*_95_ (Fig. SI10). Effects of increased relative allocation to growth of reproductive tissue are described in Note SI2.7, showing altered allometric scaling of total reproductive costs and changed growth patterns (Fig. SI11). Currently we have no experimental data to compare this model behaviour and predictions to.

## Discussion

We developed a model for ontogenetic and post-mature growth, using an energy balance to express growth as the net result of assimilation and metabolic costs for maintenance, foraging and food processing. The model (MGM) is similar in some respects to other previously presented growth models (see Note SI1) but differ in some important aspects, mainly the flexible level of details and description of maintenance. More specifically, MGM is able to capture a number of aspects that are not covered by previous common mechanistic growth models: (1) the division of maintenance costs into non-negotiable and negotiable parts, enabling hyperallometric scaling of maintenance and downregulated maintenance under food restriction; (2) costs of finding and processing food, (3) effects of body composition on costs for growth and maintenance, including differences between somatic and reproductive tissue; and (4) differentiated effects of cell growth and cell division on costs for somatic growth and maintenance.

Above, MGM has been evaluated by comparing its fit to experimental data for house crickets growing under ad libitum conditions with that of other growth models and showing how MGM behaves under food restriction. Here, we first discuss how and why the simplest relevant version of MGM manages to replicate observed growth trajectories better than the Generalized Standard Growth Model (GSGM), before discussing how MGM captures different aspects, typically not considered in other growth models.

### Model comparisons

Many growth models have been suggested over the years, some mechanistic, others more phenomenological. The Generalized Standard Growth Model (GSGM, Eq. (34), *d* ≤ 1), representing most common mechanistic growth models (AnaCat, OGM and DEB, see Note SI1.1), was not able to capture the empirical growth pattern for house crickets under ad libitum conditions, characterized by a near-symmetrical hump-shaped growth rate curve (Fig. 2). By comparing key ratios of observed growth trajectories with theoretical limits set by GSGM (Table SI4, Note SI2.1) it was demonstrated that GSGM is unable to capture the observed growth pattern, no matter how model parameters are tweaked. MGM however, can generate accurate predictions (using optimized parameters, “Numerical model comparisons”). This relies on increased relative allocation to defence (negotiable maintenance processes) during growth, resulting in maintenance costs that increase faster than linearly with body mass and consequently a rapid decline in available energy for growth. Further comparisons between MGM and different versions of GSGM (OGM and DEB) are provided in Note SI1–2.

### Effects of food restriction

The MGM parameter *δ* (the defence reduction exponent, Eqs. (25) and (29)) describes how fast negotiable maintenance costs are reduced with decreased food availability (as measured by *φ*, the relative food acquirement, Eq. (12)). With *δ* ≲ 1, MGM predictions under food restriction are (*i*) reduced ultimate body mass (*W*_*U*_) and (*ii*) reach of *W*_95_ = 0.95*W*_*U*_ at a later age *t*_*95*_, resulting in a negative correlation between *t*_*95*_ and *W*_95_ for different levels of food restriction (Fig. 3e). Such a pattern is indicated by experimental data for food-restricted cohorts of house crickets, which also display a negative correlation between age and size at maturation (Fig. 3b). There is strong empirical support for this to be a common pattern among insects and other organisms^44,45^, contrary to simple life-history theory predictions for the relationship between age and size at maturity^46,47^.

An explanation for reaction norms with earlier maturation at larger size under more favourable conditions and exceptions thereof has been suggested by Day and Rowe^48^, based on an evolutionary response to developmental thresholds (minimum sizes that organisms must reach before transition to mature state). This theoretical framework has received some empirical support^49,50^, but remains to be incorporated into dynamical growth models. A possible application of MGM is modelling of reaction norms under various environmental conditions, but this requires additional assumptions such as a response to the occurrence of developmental thresholds.

With larger *δ*, *W*_95_ under food restriction is less reduced compared to ad libitum conditions (Fig. 3e), a consequence of larger downregulation of negotiable maintenance. Increased relative allocation to growth under food restriction and reduced metabolic rate has previously been observed in moths^30^ and was interpreted as adaptive phenotypic plasticity where growth is prioritized at the expense of maintenance in order to quickly reach maturity when food conditions are poor and mortality costs for long development time are large.

The OGM for food restriction has previously been used to predict that the age when animals reach a specified fraction of the ultimate body mass is unaffected by the food availability and this was supported by data for mammals^35^. This is not generally predicted by MGM (Fig. 3f) and is not the pattern observed in house crickets (Fig. 3c). MGM predicts that animals reach a specified fraction of the ultimate body mass later at increased food restriction if relative allocation to negotiable maintenance costs increases considerably during growth (*b*_*N*_ ≳ 0.5). However, with constant relative defence allocation (*b*_*N*_ = 0), corresponding to common mechanistic growth models, the age when reaching a specified mass fraction is roughly independent on food availability, in agreement with^35^ (see Fig. SI4). Thus, MGM seems able to model a wider range of organisms under food limitation, compared to other growth models.

### Effects of feeding costs

Contrary to most previous growth models, MGM specifies feeding costs *R*_*F*_ explicitly by assuming that they increase with ingestion rate *S*. The simplest way to implement this is a proportional relationship (*R*_*F*_ = *k*_*F *_·*S*), but various complicating effects may be included, such as increased costs due to interference competition or increased foraging effort at low food availability. In MGM the extent of feeding costs in relation to other metabolic costs has considerable effects on growth rate, ultimate body size and allometric scaling of metabolic rates (Fig. SI5).

### Effects of body composition

Since most biomass is composed of similar proportions of carbohydrates, proteins and lipids, it has been suggested that the energy density of dry biomass can be considered a biological constant^51^. Nevertheless, body composition may differ between species and change during the life cycle of an individual. If required, MGM can describe effects of changes in body composition. Increased energy density of newly synthesized biomass *E*_*M*_ (e.g. as a result of maturation that involves synthesis of reproductive tissue with higher energy density) would result in decreased growth rate (Fig. SI6). In addition, MGM enables consideration of differences in growth overhead costs and maintenance costs between somatic and reproductive tissue.

### Effects of growth strategy

On a very general level, animals increase body mass mainly through cell division during early ontogeny and mainly through cell enlargement at later stages^52^ and this could have implications for metabolism^19^. Indeed, effects of cell size on metabolic rates have some empirical support for insects^53^.

Changes in number of ommatidia in the insect compound eye during ontogeny has previously been used as a proxy for the proportion of growth that is attributed to increase in cell number vs. cell size^54^. Preliminary data on house crickets (personal observations) indicate that the compound eye grows by a combination of cell division and cell enlargement, where the latter initially contributes most. This indicates a deviation from the general pattern or, alternatively, that compound eyes are not representative of other body tissues. This is well-worth to investigate closer and with more accurate and detailed data MGM is well-equipped to study consequences of growth strategies among animals. More specifically, MGM accounts for effects of growth strategy through differentiated growth overhead costs for cell division and cell enlargement combined with cell-size-dependent maintenance costs due to cost differences between surface-dependent and volume-dependent maintenance processes (Figs. SI7-8).

### Effects of allocation to negotiable maintenance costs

By the assumption of increasing relative allocation to negotiable maintenance costs during growth, MGM provides an explanation of observed growth patterns in house crickets reared at near ad libitum (Fig. 2b). Under this assumption, total maintenance rate scales super-linearly with body mass, while rates of resting and total metabolism scale sub-linearly (Fig. SI9def), in agreement with previously reported allometries for resting metabolic rate in house crickets^55,56^. Increased mass-specific costs for maintenance with increased body size makes biological sense from a number of perspectives: (1) increased priority of maintenance with increased amount of built-up capital; (2) priority of growth at early life stages; (3) increased maintenance demands due to increased complexity of tissue; and (4) increased maintenance demands due to ageing tissue. All may be contributing explanations. The available literature on regulation of maintenance generally considers immune function and these costs may be used as a proxy for negotiable maintenance costs. Energetic costs for maintenance of the immune system are known to be high, but difficult to measure^57,58^, and disease-resistance is considered to be an important life-history trait subjected to trade-offs against growth and reproductive effort^59,60^. Some empirical support for increased allocation to the immune system with age in house crickets is provided by Pinera et al.^53^. However, the variation in allocation to immune maintenance with body mass during growth needs more empirical investigation.

Depending on life history, strategies for allocation to negotiable maintenance may differ between animals. MGM can account for this by varying the relative defence allocation exponent *b*_*N*_ (Eq. 30), which may result in linear (*b*_*N*_ = 0) or hyperallometric (*b*_*N*_ > 0) scaling of maintenance (Figs. SI9-10) and by differentiating allocation to negotiable maintenance between somatic and reproductive tissue. Different allocation strategies may reflect the large variation in metabolic exponents observed among animals. MGM may thus harmonize with the suggested paradigm shift in metabolic theory from explanations based on physical constraints towards adaptive regulations^13^.

### Effects of allocation to reproduction

Larger females of fish are known to produce disproportionately larger amounts of eggs^61^, a common pattern also in other taxa^62^. Based on this, it has been proposed that hyperallometric allocation to reproduction, rather than increased maintenance costs relative to energy acquisition, explains slowed growth in animals^62^. Marshall and White^62^ obtained god fit to data for somatic growth in marine fish, applying a modified version of OGM where energy intake and expenditure scaled with the same hypoallometric exponent (< 1) and a term with hyperallometric exponent (> 1) was added to represent costs for reproductive allocation. The addition of such a term in MGM would enable good fit with data for house crickets at ad libitum, without requiring hyperallometric maintenance costs, but we consider this a non-mechanistic approach. It is not clear what the additional term represents and why it relates to body mass by a power expression. In the proposed version of MGM, growth involves both somatic and reproductive tissue (gonads, sperms, eggs, reproductive buffer). Increased allocation to reproduction is described mechanistically by increased relative growth of reproductive tissue, possibly combined with assumptions of higher energy density and/or higher specific growth overhead costs (Fig. SI11). Total reproductive growth costs are proportional to both total growth rate and an allometric factor that represents increased relative reproductive allocation, resulting in an initial hyperallometry and a final decline to zero at ultimate body size (Fig. SI11e). The corresponding growth equation is very different from the one proposed by Marshall and White^62^. It is demonstrated that hyperallometric relative reproductive growth in MGM cannot replace hyperallometric maintenance as explanation for observed growth curves in house crickets reared at near ad libitum (Note SI2.7).

After reach of ultimate body size, where assimilated energy is balanced by expenditures of feeding and maintenance in MGM, energy for offspring production is released through downregulation of negotiable maintenance costs and/or by breakdown of energy reserves.

## Conclusions

A new growth model (MGM) was developed based on an energy balance that includes several metabolic components that enable high generality and flexibility in the inclusion of various effects that may be significant, depending on organism and ecological context, many of them neglected by common mechanistic growth models. The most important contribution is the division of maintenance costs into non-negotiable and negotiable parts, enabling hyperallometric scaling of maintenance and downregulation of maintenance under food restriction. Currently, however, empirical understanding of how maintenance costs respond to variation in food availability in different organisms is poor and much more research paying attention to this important topic is needed to test our hypothesis. The model may describe various life-history dependent trade-offs between growth and negotiable maintenance, and may be used as a basis when modelling patterns at ecological levels above the individual. Here we described and derived the model, and provided some numerical simulations to demonstrate its behaviour under various circumstances. Unlike common mechanistic growth models, our model was able to capture the ad libitum growth observed in an insect. Though comparisons with data for only one species were made, MGM is believed to be a general model for individual growth and should be applicable also to other invertebrates and indefinite growers. Different developmental stages of holometabolous insects may be described using stage-specific model parameters with the transition from larval to pupal stage determined by a threshold mass. In order to thoroughly test the model, additional data will be collected from experiments on insects growing at ad libitum and food restriction.

## Methods

The basic features of the Maintenance-Growth Model (MGM) were derived in “The maintenance-growth model”. Below, individual model components of MGM are first derived and mathematical expressions for these suggested (“Derivation and specification of model components”), before detailing possible simplifications of MGM (“Possible model simplifications”). Next, the empirical data used to analyse MGM and other model approaches are described (“Empirical data from previous study”), before summarizing how model parameters were numerically optimized to data and the fit evaluated (“Numerical model comparisons”). Suggestions for more detailed descriptions of effects discussed below are found in Note SI3.

### Derivation and specification of model components

#### Ingestion rate

The ingestion rate *S* is measured as ingested energy per unit of time *t*. The maximum ingestion rate *S*_*max*_ is the ingestion rate that is realized under ad libitum conditions and is here (and often elsewhere^7^) assumed to be allometrically related to body mass *W*, at least during ontogeny:

11 $$\left\{ {\begin{array}{*{20}l} {S_{{\max }} = \alpha W^{\beta } ,} \hfill & {W \le W^{\prime}} \hfill \\ {S_{{\max }} \le \alpha W^{\beta } ,} \hfill & {W > W^{\prime}} \hfill \\ \end{array} } \right.$$

Here, *β* is an allometric exponent, *α* is a normalisation constant and *W*´ is the body mass where the allometry potentially breaks. The allometry may reflect size-dependent physical limitations of the gastrointestinal system as well as size-dependent changes in foraging behaviour with increased body mass. For indeterminate growers that grow throughout their whole lifespan (like many fishes), the allometry may very well hold generally. However, for determinate growers (like many insects), it may be the case that an animal under ad libitum conditions stops feeding at its maximum capacity at maturation or some time thereafter (*W* = *W′*). The animal may even reduce its ingestion rate below the achieved maximum level as it continues to grow.

Under food limitation, the ingestion rate is dependent on both food availability and ingestion capacity. The realized ingestion rate (*S*) may then be expressed as a proportion (*φ*) of the maximum ingestion rate (*S*_*max*_):

12 $$S = S(\varphi ,W) = \varphi S_{\max } (W),\quad \quad 0 \le \varphi \le 1$$

The ratio *φ* = *S*/*S*_*max*_ is called the *relative food acquirement*.

In some cases, the food supply is known and directly governs the food acquirement. In other cases, the food acquirement may be dependent on foraging behaviour.

#### Energy content of synthesized body tissue

Synthesized body tissue is composed of somatic tissue (with energy density *E*_*MS*_) and reproductive tissue (with energy density *E*_*MR*_). Somatic tissue may be further divided into carbohydrates/proteins (with energy density *E*_*CP*_), lipids (with energy density *E*_*L*_) and others (with zero energy density). Reproductive tissue includes gonads, sperms, eggs and possibly a reproductive buffer. The rate at which energy is bounded into synthesized biomass is:

13 $$G = E_{M} (W) \cdot \frac{dW}{{dt}},\quad \quad E_{M} (W) = E_{MS} (1 - f_{R} ) + E_{MR} f_{R} = (E_{CP} f_{CP} + E_{L} f_{L} )(1 - f_{R} ) + E_{MR} f_{R}$$

Here, *E*_*M*_(*W*) is the mass-specific energy content in body tissue, *f*_*R*_ and (1 −* f*_*R*_) are the proportions of body mass increase that are due to reproductive and somatic growth, respectively, whereas *f*_*CP*_ and *f*_*L*_ are fractions of somatic growth that is due to growth of carbohydrates/proteins and lipids (see Note SI3.1 for potential formulations of* f*_*R*_ and Note SI3.2 for suggestions of expressions for *f*_*CP*_ and *f*_*L*_). The energy densities of carbohydrates/proteins and lipids are roughly constants; *E*_*CP*_ ≈ 17 J/mg and *E*_*L*_ ≈ 34 J/mg^7^.

The average energy density of synthesized body tissue should not be confused with the average energy density of all biomass (tissue that has already been synthesized). However, with constant body composition, the two are equal and constant. *E*_*M*_ is on average 7 J/mg for fresh animal tissue^7^, but shows variation across taxa and ontogenetic stages^63^.

#### Growth overhead costs

It is assumed that growth overhead costs (*R*_*G*_) are composed of two parts; mass-specific overhead costs for producing somatic tissue (*E*_*SS*_) and reproductive tissue (*E*_*SR*_) respectively, and that potentially, specific overhead costs are different for somatic cell division (*E*_*SSD*_) and somatic cell growth (*E*_*SSG*_):

14 $$R_{G} = E_{S} (W) \cdot \frac{dW}{{dt}},\quad \quad E_{S} (W) = E_{SS} (1 - f_{R} ) + E_{SR} f_{R} = \left[ {E_{SSD} (1 - f_{G} ) + E_{SSG} f_{G} } \right](1 - f_{R} ) + E_{SR} f_{R}$$

Here, *f*_*G*_ and (1 −* f*_*G*_) are the proportions of somatic growth that are due to cell growth and cell division, respectively. See Note SI3.3 for a potential formulation of *f*_*G*_, where it increases allometrically with body size. The simplest model alternative however assumes constant *f*_*R*_ and *f*_*G*_, and thus constant specific growth overhead cost *E*_*S*_.

Costs of moulting are not explicitly treated by MGM, but can be considered a part of somatic growth overhead costs.

#### Feeding costs

Feeding costs (*R*_*F*_) include all metabolic costs for searching and processing food, more specifically costs for foraging activity (*R*_*A*_) and metabolic costs for digestion, assimilation, excretion and secretion (*R*_*D*_):

15 $$R_{F} = R_{A} + R_{D}$$

Activity costs (*R*_*A*_) are costs for activities necessary to acquire food and are assumed to generally increase with the amount of food ingested, i.e. they can be described as a monotonically increasing function of *S*:

16 $$R_{A} = R_{A} (S),\quad \quad \frac{{dR_{A} }}{dS} > 0$$

Here, *R*_*A*_ increases slowly with *S* if food is easily obtained and more steeply with *S* if food is demanding to acquire.

The digestive costs *R*_*D*_ are also assumed to increase monotonously with the ingestion rate:

17 $$R_{D} = R_{D} (S),\quad \quad \frac{{dR_{D} }}{dS} > 0$$

Here, *R*_*D*_ increases slowly with *S* if food is easily metabolized and more steeply with *S* if not.

It may not be easy to separate foraging activity costs from digestive costs. Since they relate to ingestion rate in a similar way, it may be convenient to fuse them into a single metabolic component. The feeding costs (*R*_*F*_) thus include all metabolic costs for searching and processing food and are also described as a monotonously increasing function of *S*:

18 $$R_{F} (S) = R_{A} (S) + R_{D} (S),\quad \quad \frac{{dR_{F} }}{dS} > 0$$

The simplest model version is one where feeding costs are proportional to ingestion rate:

19 $$R_{F} = k_{F} \cdot S$$

#### Maintenance costs

It is assumed that maintenance costs are composed of two main parts: ‘non-negotiable’ basal maintenance costs (*R*_*MB*_) that is a function of body size (and composition) and ‘negotiable’ costs (*R*_*MN*_) that depend on the food availability and life history strategy of the animal:

20 $$R_{M} = R_{MB} + R_{MN}$$

If somatic and reproductive tissue have different mass-specific basal maintenance costs (*γ*_*BS*_ and *γ*_*BR*_), the basal maintenance costs are divided into two parts:

21 $$R_{MB} = R_{MBS} + R_{MBR} = \gamma_{BS} W_{S} + \gamma_{BR} W_{R} = \gamma_{BS} (1 - p_{R} )W + \gamma_{BR} p_{R} W = \gamma_{B} (W) \cdot W$$

Here, *W*_*S*_ and *W*_*R*_ are somatic and reproductive body mass, whereas (1 −* p*_*R*_) and *p*_*R*_ are the proportions of total body mass that are reproductive and somatic (see Note SI3.1 for description of *p*_*R*_). If *γ*_*BS*_ and *γ*_*BR*_ are constants and *γ*_*BS*_ = *γ*_*BR*_ (or the proportions of body mass that are somatic and reproductive tissue do not change during growth), *γ*_*B*_ is a constant (and *R*_*MB*_ is proportional to body mass *W*), otherwise *γ*_*B*_ is a function of *W*. The basal maintenance costs for somatic tissue *R*_*MBS*_ may be further divided into a part that is proportional to average cell size (volume) and a part that is proportional to the average cell surface area:

22 $$R_{MBS} = R_{MBSV} + R_{MBSA} = \gamma_{BS} (W) \cdot (1 - p_{R} )W$$

A differentiation like this may be motivated by the fact that a significant amount of resting metabolism is spent on maintaining ion potentials across membranes^64^. These costs can be expected to increase with cell surface area, while other costs (oxidative processes, protein synthesis, glycogenesis etc.) can be expected to increase with cell volume. If the animal grows by a combination of cell division (increase of cell numbers) and cell growth (increase of cell size), the average cell size may change as the animal grows and thus also the mass-specific basal maintenance costs (*γ*_*BS*_).

It is assumed that ‘negotiable’ maintenance costs (*R*_*MN*_) consist of all processes that can be ‘tuned down’ by an animal to save energy. This consist mainly of ‘allocation to defence’ (maintaining the immune system and buffering against poor conditions) and ‘negotiable’ activity costs (non-necessary activity that is not directly linked to foraging activities required for attaining the actual level of ingestion). As such, these ‘negotiable’ costs (*R*_*MN*_) depend on (*i*) the food availability and (*ii*) life history strategy of the animal; the higher the level of food availability, the more energy will be available for ‘negotiable’ costs (*R*_*MN*_) and growth-related costs (*R*_*G*_ + *G*) after basal maintenance costs (*R*_*MB*_) and feeding costs (*R*_*F*_) have been paid (see Fig. 1b). How much of the total maintenance that is actually used for ‘defence’ and how much that is used for growth related costs will be determined by the life history strategy of the animal (how defence of somatic and reproductive tissue is prioritized in relation to growth at different levels of food availability). The negotiable maintenance costs (*R*_*MN*_) can be divided into somatic and reproductive parts:

23 $$R_{MN} = R_{MNS} + R_{MNR}$$

The trade-off between energy allocated to defence and energy available for growth is here described by specifying negotiable maintenance of somatic tissue (*R*_*MNS*_) as a fraction (*ρ*_*S*_) of total somatic maintenance (*R*_*MS*_) and negotiable maintenance of reproductive tissue (*R*_*MNR*_) as a fraction (*ρ*_*R*_) of total reproductive maintenance (*R*_*MR*_). The fractions are functions of relative food acquirement (*φ*) and tissue mass (*W*_*S*_ or *W*_*R*_):

24 $$\begin{gathered} R_{{MNS}} = \rho _{S} (\varphi ,W_{S} ) \cdot R_{{MS}} ,\quad \quad 0 \le \rho _{S} < 1 \hfill \\ R_{{MNR}} = \rho _{R} (\varphi ,W_{R} ) \cdot R_{{MR}} ,\quad \quad 0 \le \rho _{R} < 1 \hfill \\ \end{gathered}$$

The fractions (*ρ*_*S*_ and *ρ*_*R*_) are assumed to decrease with decreasing relative food acquirement *φ*. This can be described by the functions:

25 $$\begin{gathered} \rho _{S} (\varphi ,W_{S} ) = \rho _{{NS}} (W_{S} ) \cdot \varphi ^{\delta } ,\quad \quad 0 \le \rho _{{NS}} < 1 \hfill \\ \rho _{R} (\varphi ,W_{R} ) = \rho _{{NR}} (W_{R} ) \cdot \varphi ^{\delta } ,\quad \quad 0 \le \rho _{{NR}} < 1 \hfill \\ \end{gathered}$$

Here, *ρ*_*NS*_ and *ρ*_*NR*_ are the proportions of somatic and reproductive maintenance costs that are allocated to ‘defence’ under ad libitum conditions, and *δ* describes how fast ‘defence’ allocation is reduced with decreasing relative food acquirement. The simplest assumption is that constant proportions are allocated to negotiable maintenance costs under ad libitum conditions (*ρ*_*NS*_ and *ρ*_*NR*_ are constants). Alternatively, it can be assumed that relative defence allocation increases with the amount of produced tissue, representing a deceasing priority to growth as the animal increases in size. In line with this, we assumed that the proportions of the somatic and reproductive maintenance that are allocated to defence under ad libitum conditions are increasing with somatic and reproductive body mass according to power laws:

26 $$\rho_{NS} (W_{S} ) = a_{NS} W_{S}^{{b_{NS} }} ,\quad \quad \rho_{NR} (W_{R} ) = a_{NR} W_{R}^{{b_{NR} }}$$

Insertion of Eq. (24) into *R*_*MS*_ = *R*_*MBS*_ + *R*_*MNS*_ and *R*_*MR*_ = *R*_*MBR*_ + *R*_*MNR*_, yields negotiable maintenance costs expressed in terms of basal maintenance costs as:

27 $$R_{MNS} = \frac{{\rho_{S} }}{{1 - \rho_{S} }} \cdot R_{MBS} ,\quad \quad R_{MNR} = \frac{{\rho_{R} }}{{1 - \rho_{R} }} \cdot R_{MBR}$$

Insertion of Eq. (27) into Eq. (23), with *R*_*MBS*_ = *γ*_*BS*_*W*_*S*_ and *R*_*MBR*_ = *γ*_*BR*_*W*_*R*_, yields total negotiable maintenance costs in terms of body mass components:

28 $$R_{MN} = \frac{{\rho_{S} }}{{1 - \rho_{S} }} \cdot \gamma_{BS} W_{S} + \frac{{\rho_{R} }}{{1 - \rho_{R} }} \cdot \gamma_{BR} W_{R}$$

With Eqs. (25) and (26) inserted into Eq. (28), the total maintenance costs (*R*_*M*_ = *R*_*MB*_ + *R*_*MN*_) are obtained as:

29 $$R_{M} (\varphi ,W) = \frac{{\gamma_{BS} W_{S} }}{{1 - a_{NS} W_{S}^{{b_{NS} }} \cdot \varphi^{\delta } }} + \frac{{\gamma_{BR} W_{R} }}{{1 - a_{NR} W_{R}^{{b_{NR} }} \cdot \varphi^{\delta } }},\quad \quad \left\{ {\begin{array}{*{20}l} {W_{S} = (1 - p_{R} )W} \hfill \\ {W_{R} = p_{R} W} \hfill \\ \end{array} } \right.$$

Furthermore, due to differences in proportions of somatic and reproductive tissue, and in priority of their maintenance, the defence-growth trade-off may differ considerably between sexes of the same species (*a*_*NS*_, *a*_*NR*_, *b*_*NS*_ and *b*_*NR*_ may be sex specific parameters).

The simplest model alternative however, assumes that specific basal somatic maintenance costs are independent on cell size and type of tissue, and the same as reproductive maintenance costs (*γ*_*BS*_ = *γ*_*BR*_ = *γ*_*B*_) and that somatic and reproductive defence costs follow the same allometry (*a*_*NS*_ = *a*_*NR*_ = *a*_*N*_ and *b*_*NS*_ = *b*_*NR*_ = *b*_*N*_) so that:

30 $$R_{M} (\varphi ,W) = \frac{{\gamma_{B} W}}{{1 - a_{N} W^{{b_{N} }} \cdot \varphi^{\delta } }}$$

### Possible model simplifications

Since the mass-specific basal maintenance costs of somatic and reproductive tissue (*γ*_*BS*_ and* γ*_*BR*_), the energy density of synthesized biomass (*E*_*M*_) and the specific growth overhead cost (*E*_*S*_) may change during growth due to changes in body composition and/or type of growth at the cellular level, we have here defined them generally as functions of body mass to allow as much biological detail as needed to be included. This unavoidably leads to a complex model with many parameters. However, the parts of the model can be significantly simplified, as indicated in each section above, and are summarized here to collectively result in the simplest relevant version of MGM under the following assumptions:

First, if basal somatic maintenance costs are independent on cell size and type of tissue (somatic vs. reproductive), *γ*_*BS*_ = *γ*_*BR*_ = *γ*_*B*_ is constant. Second, if the compositions of somatic and reproductive tissue (carbohydrates, proteins, lipids) are similar and constant, *E*_*M*_ is constant. Third, if growth overhead costs are independent on growth strategy (cell division vs. cell growth) and type of tissue, *E*_*S*_ is constant. Fourth, if reproductive body mass is negligible in comparison to somatic body mass (*W*_*R*_ ≈ 0, *W*_*S*_ ≈ *W*), negotiable maintenance costs include only somatic tissue (*R*_*MN*_ ≈ *a*_*N*_*Wb*_*N*_·*φ*^*δ*^ ·*R*_*M*_). Finally, by also assuming that the maximum ingestion rate is fully described by an allometric relation (*S* = *φαW*^*β*^) and that feeding costs are proportional to ingestion rate (*R*_*F*_ = *k*_*F*_·*S*), the general growth model (Eq. 10) of MGM simplifies into:

31 $$\frac{dW}{{dt}} = \frac{1}{{E_{M} + E_{S} }}\left[ {(e - k_{F} )\varphi \alpha W^{\beta } - \frac{{\gamma_{B} \cdot W}}{{1 - a_{N} W^{{b_{N} }} \cdot \varphi^{\delta } }}} \right]$$

Furthermore, with *b*_*N*_ = 0 (relative allocation of total maintenance to negotiable costs is independent of body mass) and fixed relative food acquirement *φ*, the growth equation is a mechanistically based equivalence to that of von Bertalanffy^38^:

32 $$\frac{dW}{{dt}} = aW^{\beta } - bW\quad \quad ,\quad \quad \left\{ {\begin{array}{*{20}l} {a = (e - k_{F} )\varphi \alpha /(E_{M} + E_{S} )} \hfill \\ {b = {{\gamma_{B} } \mathord{\left/ {\vphantom {{\gamma_{B} } {\left[ {(E_{M} + E_{S} )(1 - a_{N} \varphi^{\delta } )} \right]}}} \right. \kern-0pt} {\left[ {(E_{M} + E_{S} )(1 - a_{N} \varphi^{\delta } )} \right]}}} \hfill \\ \end{array} } \right.\quad$$

This form of the MGM growth equation is compared term by term with the standard DEB growth equation in Note SI1.3.

### Empirical data from previous study

In order to compare model predictions under ad libitum conditions and illustrate the behaviour of MGM under food restriction, we used experimental data from a previous study on house crickets^42^, where cohorts of varying starting densities (newly hatched nymphs, *W*_0_ ≈ 0.67 mg) were provided a fixed amount of food at regular time intervals in order to investigate self-thinning (decreasing cohort size along with increasing average body mass due to individual growth). Individuals were regularly weighed during the experiment, which was terminated after they reached sexual maturation but well before the start of female egg production (see Jonsson^42^ for details on the experimental setup). Using these data, for each of five different starting densities (5, 10, 20, 40 and 80 individuals), we calculated the average body mass *W* (for all individuals in all cohorts of current density) for different ages *t*. The average growth rate *dW*/*dt* at these ages was, for each starting density, linearly interpolated from difference ratios applied to the average *W*/*t* data. The calculated data, representing average trends across individuals of a starting density, were used to plot empirical growth curves. Average age and body mass at maturation (imago emergence) for cohorts of different starting densities were calculated for each sex separately. Smooth curves were generated from quadratic fits to non-averaged empirical data of growth rate vs. body mass (MATLAB routine *polyfit*), combined with estimated average data for high density cohorts (departing considerably from a quadratic fit at late ages). These were used to identify maximum growth rate ${\dot{W}}_{max}$ and corresponding body mass W^*^, and to generate smooth growth curves (*W* vs. *t* with MATLAB ODE solver *odes23*). The latter were used to identify the ultimate body mass *W*_*U*_ and ages at reach of different specified fractions of *W*_*U*_. In MGM and other analysed growth models, *W*_*U*_ is an asymptote (approached when *t* → ∞), and thus *t*_95_ was used as a measure of age at final body size. The starting density of five individuals represents near ad libitum conditions and data from these cohorts were used to study predictions of MGM and other models under no food limitation. For near ad libitum cohorts, identified growth properties were used to calculate the dimensionless key properties ω* and Ω (see Note SI2.1 and Table SI4).

### Numerical model comparisons

The simplified version of MGM in Eq. (31) is further simplified under ad libitum conditions (*φ* = 1) and linear relative defence allocation (*b*_*N*_ = 1):

33 $$\frac{dW}{{dt}} = aW^{\beta } - \frac{cW}{{1 - a_{N} W}}\quad ,\quad \left\{ {\begin{array}{*{20}l} {a = (e - k_{F} )\alpha /(E_{M} + E_{S} )} \hfill \\ {c = \gamma_{B} /(E_{M} + E_{S} )} \hfill \\ \end{array} } \right.$$

Four free model parameters then remain (*a*, *β*, *c*, *a*_*N*_). This version of MGM was compared with the Generalized Standard Growth Model (GSGM), representing common mechanistic growth models (AnaCat, OGM and DEB, see Note SI1.1) using three free model parameters (*a*, *b*, *c*) and *d* = 1 (required for best data fit):

34 $$\frac{dW}{{dt}} = aW^{b} - cW^{d}$$

A comparison with the logistic growth model^43^ and a growth model by Makarieva, et al.^37^ are also included in Note SI2.1.

For each model type, parameters were optimized (Table SI3) using the ‘inverse method’^65,66^ that minimized least squares between model prediction and averaged data (house crickets growing under near ad libitum conditions^42^). All analyses were performed with the software MATLAB^®^ (version R2021a, Mathworks Inc., Natick, MA, USA), including use of the numerical optimization function *fmincon* and ODE solver *ode23s*.

Goodness of fit (between predicted and empirical averaged growth curve) was quantitatively evaluated by the measure:

35 $$GF = 1 - \frac{{\sqrt {\sum\nolimits_{i = 1}^{n} {(O_{i} - E_{i} )^{2} } } }}{{\sqrt {\sum\nolimits_{i = 1}^{n} {(O_{i} - \overline{O})^{2} } } }},\quad \quad \overline{O} = \frac{1}{n}\sum\nolimits_{i = 1}^{n} {O_{i} }$$

Here *O*_*i*_ and *E*_*i*_ are observed and predicted value of data point *i* and *n* is number of observations. Each data point represents the average mass of all individuals (in all near ad libitum cohorts) of a certain age. A perfect fit results in the maximum value *GF* = 1. The closer *GF* is to unity, the better is the fit.

### Supplementary Information

Supplementary Information.

## Data Availability

The datasets used and analysed during the current study are available from the corresponding author on reasonable request.
