# Author Correction: A new flexible model for maintenance and feeding expenses that improves description of individual growth in insects

**DOI:** 10.1038/s41598-023-45923-5

**Published:** 2023-11-01

**Authors:** Karl Mauritsson, Tomas Jonsson

**Affiliations:** 1 Ecological Modelling Group, School of Bioscience, University of Skövde, Skövde, Sweden; 2 Ecological and Environmental Modeling, Department of Physics, Chemistry and Biology, Linköping University, Linköping, Sweden

Correction to: *Scientific Reports* 10.1038/s41598-023-43743-1, published online 05 October 2023

The original version of this Article omitted an affiliation for Karl Mauritsson and Tomas Jonsson. Their correct affiliations are listed below.

Ecological Modelling Group, School of Bioscience, University of Skövde, Sweden.

Ecological and Environmental Modeling, Department of Physics, Chemistry and Biology, Linköping University, Sweden.

The original Article has been corrected.

