# Supplementary material for: A new flexible model for maintenance and feeding expenses that improves description of individual growth in insects
Source: Sci Rep. 2023 Oct 5;13:16751. doi: 10.1038/s41598-023-43743-1 (PMC10556006; doi:10.1038/s41598-023-43743-1)
Supplement: Supplementary file 1 — Supplementary Information. [file 41598_2023_43743_MOESM1_ESM.pdf]

Supplementary Information for

A new flexible model for maintenance and feeding expenses that improves description of individual growth in insects

Karl Mauritsson, Tomas Jonsson

This supplemental information includes the following:

Note SI1 - Growth models based on metabolic theories

Note SI2 - Numerical model comparisons and demonstrations

Note SI3 - Inclusion of additional effects in developed growth model

Tables SI1-SI8

Figs. SI1-SI11

1. Growth models based on metabolic theories

1.1 Overview of common growth models

Most growth models describe how growth rate v depends on current body mass W and in a few cases, growth rate is also explicitly dependent on time t [e.g. 1,2]. The mathematical description is often a differential equation, called the *growth equation*:

$$v = \frac{dW}{dt} = f(W,t) \tag{SI1}$$

The solution to the growth equation yields the growth curve; body mass as a function of time $W(t)$. Phenomenological growth models, like the logistic growth model [3] and the famous Gompertz growth model [4], propose mathematical functions that can provide a good fit to empirical curves. However, they provide little mechanistic insight into affecting factors during growth. Mechanistic growth models instead are based on underlying mechanistic assumptions combined with an energy or mass balance that describes growth as the net result of supply and cost:

Growth = Supply – Cost

Three major groups of mechanistic growth models can be distinguished based on how the cost and supply terms are described:

1) *Anabolism-Catabolism based* (AnaCat) *growth models* describe growth as the net result of anabolism (synthesis of new biomass) and catabolism (break-down of current biomass). One of the first mechanistic growth models, proposed by Pütter [5], belongs to this category and describes growth as the net result of surface-related anabolism and volume-related catabolism. The famous von Bertalanffy (vB) growth model [6] describes anabolism as an allometric relation with flexible power exponent and catabolism as proportional to current body mass, treating allometric constants as approximation parameters without physiological interpretation. The gill-oxygen limitation (GOL) model [7] is a minor modification of the Pütter model, describing anabolism as limited by the surface involved in oxygen uptake in water-breathing animals.

2) The *Dynamic Energy Budget* (DEB) *theory*, developed by Kooijman [8], has been extensively used in theoretical ecology to predict life histories under different conditions [9]. DEB models describe the rates at which energy from ingested food is assimilated and used for maintenance, growth, development and reproduction by a system of differential equations for changes of state variables of individual organisms [10]. Metabolism is an indirect outcome of maintenance, growth and maturation, and allometric scaling of metabolic rate depends on the relative contribution from processes that are proportional to surface area and to 'structural volume'. Normally, *intra*specific metabolic scaling during ontogeny with a 2/3 power exponent (β in Eq. (1)) arises in DEB due to an increasing proportion of 'reserve' along with a decreasing proportion of energy-demanding 'structure', while *inter*specific

sub-linear allometric scaling ($\beta < 1$) arises since fully-grown individuals of larger species have larger reserve and less structure with high metabolic activity [11]. DEB *growth models* [8,12] build on DEB theory and describe growth as the net result of assimilation and maintenance. Energy from ingestion (dependent on structural surface area and food availability) is assimilated into reserve (with some losses through excretion). A fixed fraction of energy mobilized from reserve is spent on somatic maintenance (proportional to the structural volume) and growth (increase of structural volume), whereas the rest is spent on maturation and maturity maintenance or reproduction and maturity maintenance. Anabolic and catabolic processes are indirectly accounted for in the sense that ingested food is assimilated into reserve, broken down via reserve mobilisation and converted into structure (growth). Heat losses (respiration) occur from maintenance processes and growth overhead costs. The growth equation is usually expressed in terms of the structural volume (not the body mass), but under constant structural density, constant food availability and constant energy density of reserves, the biomass density is constant and body mass is proportional to structural volume.

3) *Ontogenetic Growth Models* (OGM) [13-18] describes growth as the net balance between anabolism (total metabolism) and maintenance, with total metabolic rate described by a power law in accordance with MTE (Metabolic Theory of Ecology). OGM has been updated and modified a number of times to deal with inconsistencies or include additional effects (temperature dependence and food restriction).

Under ad libitum conditions (and constant body density in DEB), the growth equations for all major groups of mechanistic growth models (AnaCat, DEB, OGM) have the same general structure, with supply and cost terms described by allometric relations (Table SI1):

$$\frac{dW}{dt} = aW^{b} - cW^{d} \qquad \text{(SI2)}$$

We call this the Generalized Standard Growth Model (GSGM). All model parameters (a, b, c, d) are positive constants. An essential feature of this growth equation is that $b < d$. The supply term (aW^b) initially increases faster with body mass than the cost term (cW^d), but the pattern is reversed after reach of maximum growth rate. Growth occurs as long as the supply term exceeds the cost term (and ceases when $aW^b = cW^d$). The growth equation thus describes a hump-shaped curve and the growth curve $W(t)$ is sigmoidal, eventually approaching the asymptotic (ultimate) body mass $W_U = (a/c)^{1/(d-b)}$ (corresponding to zero growth rate). It should be noticed that all standard mechanistic growth models (AnaCat, DEB, OGM) apply $d \leq 1$ (with $d = 1$ for all models, except GOL).

Table SI1: Major groups of mechanistic growth models under ad libitum conditions. Growth is the net result of supply and cost, both described by allometric relations (Eq. (SI2)).

**Model type**	**Supply** (aW^b)	**Cost** (cW^d)	b	d
AnaCat	Anabolism	Catabolism	2/3 (Pütter)<br>adjustable (vB & GOL)	1 (Pütter & vB)<br>≤ 1 (GOL)
DEB	Assimilation	Maintenance	2/3	1
OGM	Anabolism	Maintenance	3/4	1

In DEB, species with different ultimate body mass have different allometric relations for assimilation rate (different a). In OGM, species with different ultimate body size have different mass-specific maintenance rates (different c). Though the underlying assumptions are different (Table SI1), all common mechanistic models make similar predictions under ad libitum conditions. Model parameters may in fact be adjusted in a way such that growth curves become almost indistinguishable. Predictions differ when different life stages and variable environmental conditions (e.g. varying food availability) are considered.

1.2 Growth models for food restriction

A growth model useful for metabolic theory, must be able to deal with limited resource availability and its effect(s) on metabolism and growth. It is well known that food restriction lowers resting metabolic rates in growing animals [19,20], including insects [21], fish [22], birds [23] and mammals [24]. This is partly a consequence of less energy available for growth, resulting in decreased growth rates and accordingly, decreased growth overhead costs [25]. In some animals, there is evidence also for downregulation of maintenance processes under food restriction, believed to be an adaptive response that favours growth in insects [26] and birds [27-29] or avoids weight loss in fish [22]. Furthermore, many insects respond to food limitation by lowered maintenance of the immune system [30]. On the other hand, there is empirical support that food restriction may result in upregulation of cellular recycling and repair mechanisms, which has been linked to increased lifespan in rodents [31,32]. In general, fast growth has negative effects on maintenance [33,34], immune function [35], performance [36] and life span [37]. Obviously, there is a trade-off between growth and maintenance [38]. Species with long life spans generally maximize fitness by prioritizing maintenance at the expense of growth in order to maintain good health during periods of food restriction, while species adapted to complete their life cycle before seasonal periods of food shortage generally maximize fitness by prioritizing growth at the expense of maintenance metabolism [26].

Food-limitation is not explicitly accounted for in AnaCat models, but parameter values may be altered in order to adjust for changes in anabolic and catabolic rates.

The OGM for food restriction [13], a development of previous versions of OGM [14,15], describes energy assimilated from food as distributed between total metabolism (activity and resting metabolism) and energy that is bounded into newly synthesized biomass. Growth overhead costs and somatic maintenance are included as parts of resting metabolism. This is in line with the energy balance of the Maintenance-Growth Model (MGM), the new model proposed here, but OGM does not account for metabolic costs of food processing. OGM also diverges from MGM by assuming that resting and total metabolic rate are proportional, an assumption that lacks mechanistic foundation and is incompatible with regulation of maintenance in response to food limitation (as suggested by MGM). In OGM, resting and total metabolic rates are described by allometric relations with constant power exponents and normalisation constants that are reduced in proportion to the reduction of assimilation under food restriction. Growth rate and ultimate body mass are reduced as a consequence of less metabolic energy available. The OGM for food restriction has obtained successful predictions for mammals and birds [13], but has as far as we know not previously been applied to insects.

In DEB models [12] reduced growth rate and ultimate body mass are direct consequences of reduced assimilation under food limitation, resulting in less energy available for growth. Resting metabolic rate is reduced as a consequence of reduced growth overhead costs. Growth rate is also reduced under poor nutritional status (due to previous food limitation), since assimilation and maintenance depends on reserve density [39].

Finally, a fourth type of model for food-restricted growth, developed by Makarieva, et al. [40] describes assimilation (A) as distributed between resting metabolism (R_R) and growth expense (G), $A = R_R + G$, assuming that growth expense is a specified fraction of the assimilation rate, $G = \varepsilon \cdot A$. The fraction ε (called *growth efficiency*) is a function of body mass and takes different shapes depending on food conditions, where one option is a linearly decreasing growth efficiency with body mass, $\varepsilon = \varepsilon_0 (W_U - W)$, reaching zero at ultimate body mass (W_U). The growth expense considered is energy bounded into newly synthesized biomass, $G = E_M \cdot dW/dt$. Resting metabolism is described by an allometric relation ($R_R = a_R W^b$). These assumptions yield the following growth equation:

$$\frac{dW}{dt} = \frac{\varepsilon}{1-\varepsilon} aW^b = \frac{\varepsilon_0 (W_U - W)}{1-\varepsilon_0 (W_U - W)} aW^b \qquad , \qquad a = a_R / E_M \tag{SI3}$$

Obviously, the model neglects growth overhead costs and activity expenses. It cannot be written in the general form of GSGM (Eq. (SI2)), i.e. as a difference between supply and cost with the allometric exponent for supply, b, being smaller than the allometric exponent for cost, d.

1.3 THEORETICAL COMPARISON OF MGM AND DEB

DEB theory divides the body into structure and reserve, where only structure requires maintenance to a cost that is proportional to the structural volume. Like MGM, DEB theory thus is able to deal with changing mass-specific costs for maintenance and growth due to changing body composition. Since animals generally increase their proportion of reserve as they grow, the mass-specific maintenance cost generally decreases during growth in DEB models [11], the opposite pattern to what MGM predicts for insects. In the standard DEB model [8], a fixed fraction (κ) of mobilized reserve energy is spent on somatic maintenance and growth, whereas the rest (1- κ) is allocated to maturation, maturity maintenance and reproduction. This does not capture how the proportion of assimilated energy that is available for growth decreases faster than linearly with body mass, as indicated by data for house crickets under (or very near) ad libitum conditions [41]. However, by allowing κ to increase with body mass, such a growth pattern may be captured by a DEB model.

The standard DEB model [8] under constant food density and neglected costs for heating and osmoregulation (surface-dependent maintenance) yields a growth equation expressed in the structural volume V:

$$\frac{dV}{dt} = \frac{1}{\kappa[E_R]+[E_G]}\left[f\kappa\{A_{max}\}V^{2/3} - [R_{MV}]V \right] \qquad \text{(SI4)}$$

Here, f is the scaled functional response (dependent on food density), $\{A_{max}\}$ is the maximum surface-specific assimilation rate, $[R_{MV}]$ is volume-specific somatic maintenance, $[E_R]$ is volume-specific reserve energy content and $[E_G]$ is volume-specific energetic growth cost. The parameters κ, $\{A_{max}\}$, $[R_{MV}]$ and $[E_G]$ are all constants in the standard DEB model and with constant food density, also f and $[E_R]$ are constants. The actual body volume is the structural volume V, but actual body mass W is the sum of structural mass and reserve mass:

$$W = \rho_S V + ([E_R]/\mu_R)\cdot V \qquad \text{(SI5)}$$

If the structural density ρ_S and the energy density of reserves μ_R both are constants, body mass is proportional to structural volume ($W = \rho V$), i.e. the biomass density ρ is constant. The standard DEB growth equation (Eq. (SI4)) can then be written in terms of body mass:

$$\frac{dW}{dt} = \frac{1}{\kappa E_R + E_G}\left[f\kappa\frac{\{A_{max}\}}{\rho^{2/3}}W^{2/3} - \gamma W \right] \qquad \text{(SI6)}$$

Here, γ is mass-specific somatic maintenance, E_R is reserve energy content per unit body mass and E_G is mass-specific energetic growth cost.

A simplified version of the MGM growth equation (Eq. (10)) under fixed relative food acquirement φ is obtained by assuming allometric ingestion rate ($S = \alpha W^{\beta}$), linear feeding costs ($R_F = k_F S$), constant biomass energy density E_M, constant specific growth overhead costs E_S, constant basal maintenance rate γ_B and undifferentiated tissue ($W_S = W$, $W_R = 0$, $a_{NS} = a_{NR} = a_N$):

$$\frac{dW}{dt} = \frac{1}{E_M + E_S}\left[\varphi(e - k_F)\alpha W^{\beta} - \frac{\gamma_B W}{1 - a_N W^{b_N} \varphi^{\delta}}\right] \tag{SI7}$$

There are both similarities and differences between the simplified MGM growth equation (Eq. (SI7)) and the standard DEB growth equation (Eq. (SI6)). MGM's total specific growth cost ($E_M + E_S$), including both energy density of synthesized biomass (E_M) and specific growth overhead costs (E_S) corresponds mathematically to the denominator in the DEB equation ($\kappa E_R + E_G$), including both costs of mobilizing energy from reserve and direct growth costs. MGM's specific growth overhead costs (E_S) may be considered as the sum of mobilisation costs and succeeding overhead costs for biomass synthesis. In MGM, E_M and E_S do not have to be constant when food availability and energy density of reserves are constant (contrary to DEB).

MGM's food availability parameter φ is similar to DEB's scaled functional response f. The ingestion rate allometric exponent β is adjustable in MGM, but is fixed to 2/3 in DEB. The product $(e\text{-}k_F)\alpha$ in MGM corresponds mathematically to the product $\kappa\{A_{max}\}/\rho^{2/3}$ in DEB. In MGM $(e\text{-}k_F)\alpha$ can be interpreted as a size-specific effective assimilation rate accounting for nutrient assimilation efficiency (e), costs of finding and processing food (k_F) and size-specific ingestion capacity (α). Correspondingly, in DEB, $\kappa\{A_{max}\}/\rho^{2/3}$ can be interpreted as a size-specific effective assimilation rate accounting for nutrient assimilation efficiency and surface-specific ingestion capacity ($\{A_{max}\}/\rho^{2/3}$) as well as life-history-dependent allocation of total available reserve energy to somatic maintenance and growth (κ), opposed to allocation for maturation and reproduction. In MGM, $\gamma_B/(1\text{-}a_N W^{b_N}\varphi^{\delta})$ is the mass-specific maintenance rate, including both basal and negotiable costs. It corresponds to the mass-specific somatic maintenance γ in DEB, which in contrast to MGM is independent of body mass W and food availability φ (as long as food density and energy density of reserves are constant). However, with $b_N = 0$ (constant relative allocation to negotiable maintenance costs), also MGM's mass-specific maintenance rate is independent of body mass. To summarize, DEB includes effects of life-history dependent allocation in the source term occurring from the so called κ-rule [8], whereas MGM includes effects of life-history dependent allocation in the cost (maintenance) term.

2. Illustration of MGM behaviour and comparisons with other model approaches

MGM (Eq. (10)) is in this contribution analysed under a number of scenarios. In the main text the general behaviour of MGM under 1) ad libitum conditions (and a number of simplifying assumptions, main section 3.1) and 2) food restriction (main section 3.2), is illustrated by numerical predictions and comparison with other growth models and data from a previous study on house crickets [41]. More details on these analyses can be found below (Note SI2.1-2). In addition, the behaviour of MGM under five specific ad libitum scenarios are investigated and illustrated below (Note SI2.3-7), including effects of (3) feeding costs, (4) body composition, (5) growth strategy, (6) allocation to negotiable maintenance and (7) allocation to reproduction. The aim of this note is to shed more light on how different components of MGM, such as maintenance and feeding costs, respond and interact to produce MGM behaviour in various situations.

All MGM simulations presented here assumed an allometric relation for maximum ingestion rate, linear feeding costs (Eq. (19)) and undifferentiated maintenance costs for somatic and reproductive growth (Eq. (30)), simplifying Eq. (10) into:

$$\frac{dW}{dt} = \frac{1}{E_M + E_S}(eS - R_F - R_M) \quad , \quad \begin{cases} S = \varphi\alpha W^{\beta} \\ R_F = k_F S \\ R_M = \gamma_B W / (1 - \rho_N \varphi^{\delta}) \quad , \quad \rho_N = a_N W^{b_N} \end{cases} \tag{SI8}$$

Body mass W versus age t was obtained by solving this differential equation numerically. Predictions of resting (R_R) and total metabolic rate (R_{tot}) were then calculated as:

$$R_R = R_G + R_M = E_S \tfrac{dW}{dt} + R_M \qquad , \qquad R_{tot} = R_R + R_F \tag{SI9}$$

Approximate allometric exponents of metabolic rates were estimated as slopes of straight lines fitted to logarithmic data on metabolic rate vs. body mass.

Most simulations (but not all, see Note SI2.4-7) assumed constant biomass energy density (E_M), constant specific growth overhead cost (E_S), constant specific basal maintenance cost (γ_B) and linear relative defence allocation ($b_N = 1$), where values of these and other parameters (Table SI2) were chosen based on previous studies [41-43] to reflect ‘realistic’ parameter values for house crickets (*Acheta domesticus*). These preliminary parameters are only used for demonstrative purpose and may change considerably when calibrated to sufficiently detailed empirical data.

Table SI2: MGM parameter values applied in numerical demonstrations of model behaviour.
* signifies parameters for which values were varied in some cases (stated below when applicable).
- denotes parameters that are dimensionless.

Parameter	Notation	Value	Unit
Assimilation efficiency	e	0.75	-
Biomass energy density*	E_M	6.0	J/mg
Specific growth overhead cost*	E_S	3.0	J/mg
Ingestion rate normalisation constant	α	10	$J/(mg^{\beta}\cdot day)$
Ingestion rate allometric exponent	β	0.75	-
Specific basal maintenance cost*	γ_B	0.40	$J/(mg\cdot day)$
Relative defence allocation constant*	a_N	$1\cdot10^{-3}$	mg^{-1}
Relative defence allocation exponent*	b_N	1	-
Defence reduction exponent*	δ	1	-
Feeding cost slope*	k_F	0.40	-
Birth mass	W_0	1	mg
Maturation mass*	W_{mat}	300	mg
Relative food acquirement	φ	1	-

2.1 NUMERICAL COMPARISONS UNDER AD LIBITUM CONDITIONS

In main section 3.1, optimized predictions of a simplified version of the MGM growth model (Eq. (33)) and the Generalized Standard Growth Model (GSGM, Eq. (SI2), representing AnaCat, DEB and OGM, see Note SI1.1), were compared with empirical growth curves for house crickets reared under near ad libitum conditions [41]. Below, we also include the logistic growth model [3] and the Makarieva growth model (Eq. (SI3)) in a more detailed comparative analysis (Fig. SI1).

Empirical growth curves were well captured by MGM, but good agreement was not possible to obtain using GSGM (Fig. SI1a, Table SI3). More specifically, GSGM was unable to capture the near-symmetrical hump-shaped curve that characterizes growth rate as a function of body mass, peaking at a relatively large body mass compared to ultimate size (Fig. SI1b). A logistic growth model follows the mathematical form of GSGM, but with $b = 1$ and $d = 2$ (as opposed to $d \leq 1$ for GSGM) and captures empirical curves better (Fig. SI1, Table SI3), but lacks mechanistic foundation. Empirical curves are considerably better captured by the Makarieva growth model (Fig. SI1, Table SI3), but the model is believed to neglect some important metabolic components (see Note SI1.2). Finally, empirical curves are even better captured by MGM (Fig. SI1, Table SI3).

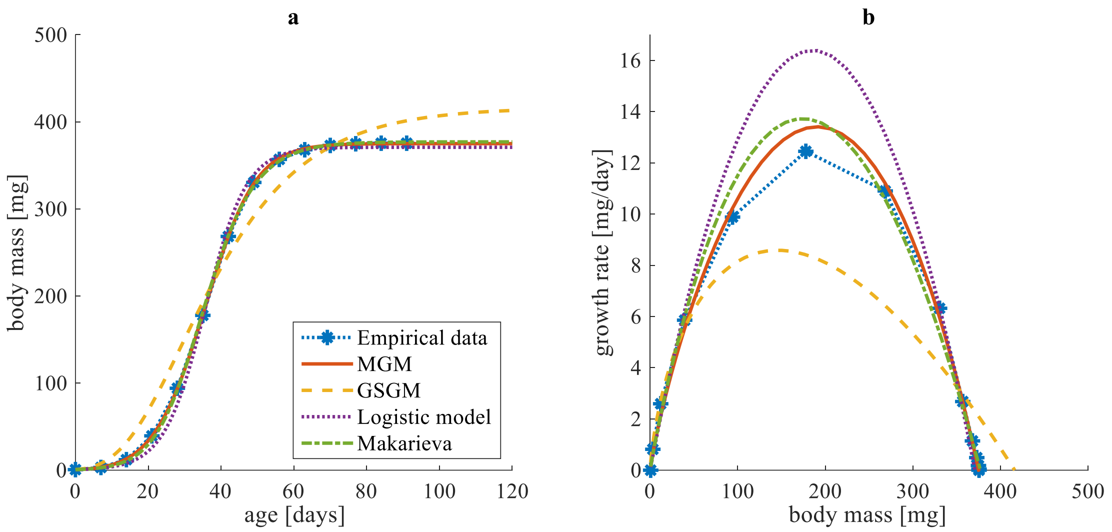

Fig. SI1: Empirical growth curves for house crickets reared under near ad libitum conditions [41] and growth curves predicted by different growth models with application of optimized parameter values (Table SI3).

Table SI3: Optimized values of model parameters and corresponding goodness of fit measures (*GF*, Eq. (35)) for the simplest relevant version of MGM (Eq. (33)), GSGM (Eq. (SI2)), the logistic model (Eq. (SI2); b = 1, d = 2) and the Makarieva model (Eq. (SI3)). For GSGM, d = 1 was applied, since worse agreement is obtained with smaller d.

**Model**	**Optimized parameter values**	**GF**
MGM	a = 0.50 mg/(day·mg$^{\beta}$)] , β = 0.94, c = 0.25 day^{-1} , a_N = 7.4·10^{-4} mg^{-1}	0.995
GSGM (d = 1)	a = 1.0 mg/(day·mg^{b})] , b = 0.91 c = 0.57 day^{-1}	0.845
Logistic model (b = 1, d = 2)	a = 0.18 day^{-1} c = 4.8·10^{-4} (mg·day)$^{-1}$	0.950
Makarieva	a = 1.0 mg/(mg^{b}·day) , b = 0.95 W_U = 377 mg , ε_0 = 4.6·10^{-4} mg^{-1}	0.985

The inability of GSGM to capture the rapid growth of insects such as house crickets at near ad libitum [41] can be further demonstrated by comparing theoretical limits of two dimensionless key properties of growth trajectories between GSGM predictions and empirical data (Table SI4). From GSGM (Eq. (SI2)), analytical expressions can be derived for ultimate body mass W_U (the asymptote $W_U = (a/c)^{1/(d-b)}$ at $dW/dt = 0$), maximal growth rate $\dot{W}_{max}$ (the growth rate at $d^2W/dt^2 = 0$) and corresponding body mass $W^* = [ab/(cd)]^{1/(d-b)}$. The relative body mass at maximum growth rate is by GSGM predicted to:

$$\omega^* = W^*/W_U = (b/d)^{1/(d-b)} \qquad \text{(SI10)}$$

The largest possible ω^* predicted by GSGM is obtained with the largest possible d and b. Since GSGM requires $b < d \leq 1$, it is obtained as:

$$\omega^*_{\max} = \lim_{b \to 1} [b^{1/(1-b)}] = 1/e \approx 0.37 \quad \text{(SI11)}$$

Consequently, GSGM cannot predict ω^* larger than 0.37, but experimental data for house crickets yield $\omega^* \approx 0.49$ (representing a growth rate curve that peaks for a relatively large body mass compared to ultimate size). As illustrated by Fig. SI2a, GSGM predictions of ω^* (with $0 \leq b \leq 1$) cannot reach the empirical value of ω^* as long as $d \leq 1$. A logistic growth model (b = 1 and d = 2) yields a parabolic curve with $\omega^* = 0.50$, which is close to experimental data.

With d = 1 (best fit option) and known relative birth mass $\omega_0 = W_0/W_U$, Eq. (SI2) can be solved analytically and an expression can be derived for age t_ω at which any relative body mass $\omega = W/W_U$ is reached. The relative maximum growth rate (Ω) relates maximal growth rate ($\dot{W}_{max}$) to mean growth rate (W_U/t_{95}):

$$\Omega = \frac{\dot{W}_{\max}}{W_U / t_{95}} = \frac{1}{1-b}\left[b^{b/(1-b)} - b^{1/(1-b)}\right]\cdot\left[\ln(1-\omega_0^{1-b}) - \ln(1-0.95^{1-b})\right] \quad \text{(SI12)}$$

Experimental data (Table SI4) yield $\log_{10}(\omega_0) \approx -2.79$ and $\Omega \approx 1.79$ for house crickets, which requires $b \approx 0.57$ for a GSGM prediction to fit (Fig. SI2b), far from commonly applied exponents ($2/3 \leq b < 1$). A summarized comparison of key growth-related properties (Table SI5) between empirical estimations ($\omega^* \approx 0.49$, $\Omega \approx 1.79$) and model predictions (using optimized parameter values, Table SI3) indicates good prediction by Makarieva ($\omega^* \approx 0.46$, $\Omega \approx 2.09$), better prediction by MGM ($\omega^* \approx 0.51$, $\Omega \approx 1.98$) and less good prediction by GSGM ($\omega^* \approx 0.35$, $\Omega \approx 1.75$).

Table SI4: Key growth-related properties calculated from empirical data of house crickets growing under near ad libitum conditions [41].

Parameter	Notation	Value	Unit
Birth mass	W_0	0.67	mg
Body mass at maximal growth rate	W^*	201	mg
Ultimate body mass	W_U	409	mg
Maximal growth rate	$\dot{W}_{max}$	13	mg/day
Age at reach of $0.95W_U$	t_{95}	57	days
Relative birth mass	$\omega_0 = W_0/W_U$	$1.6 \cdot 10^{-3}$	-
Logarithm of relative birth mass	$\log_{10}(\omega_0)$	-2.79	-
Relative body mass at maximal growth rate	$\omega^* = W^*/W_U$	0.49	-
Relative maximal growth rate	$\Omega = \dot{W}_{max}/(W_U/t_{95})$	1.79	-

Table SI5: Key growth-related properties (ω^*, Ω) obtained from data [41] and model predictions with optimized parameter values (Table SI3).

	Data	MGM	GSGM	Logistic	Makarieva
ω^*	0.49	0.51	0.35	0.50	0.46
Ω	1.79	1.98	1.75	2.31	2.09

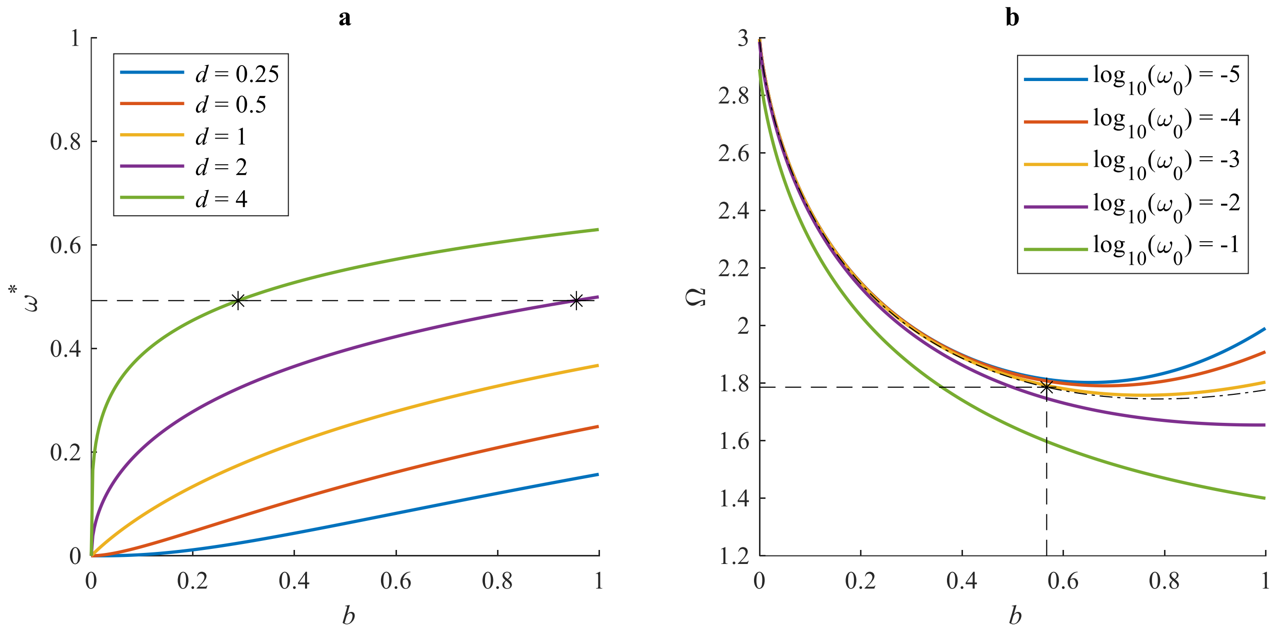

Fig. SI2: Dimensionless key properties of growth trajectories predicted by GSGM (Eq. (SI2)) as a function of *b* (allometric exponent of source term in Eq. (SI2), restricted to the range [0 1]). **a**) Relative body mass at maximum growth rate (ω^*, Eq. (SI10)) for different values of *d* (allometric exponent of cost term in Eq. (SI2)). Stars indicate fits between empirical value of ω^* (= 0.49, horizontal dashed line) and model predictions for two values of *d*. **b**) Relative maximum growth rate (Ω, Eq. (SI12)) for different values of relative initial birth mass (ω_0) with $d = 1$. Dash-dotted curve represents relative maximum growth rate (Ω) as a function of *b*, for empirical value of $\log_{10}(\omega_0)$ (= -2.79) and star indicates fit between this and empirical value of Ω (= 1.79, horizontal dashed line). Dashed vertical line thus indicates value of *b* (= 0.57) needed in GSGM to obtain empirical value of Ω for empirical value of $\log_{10}(\omega_0)$.

2.2 EFFECTS OF FOOD RESTRICTION

In MGM simulations of food restriction, fixed values of the relative food acquirement φ were applied. Some predictions of MGM as affected by various constant relative food acquirements φ were shown in main section 3.2 (Fig. 3). More details are presented here (Fig. SI3). In these simulations (using δ = 1), ultimate body mass (Fig. SI3a) and growth rates (Fig. SI3bc) are reduced with increased level of food restriction. Effects of food restriction on metabolic rates are considerable with downregulation of maintenance (Fig. SI3d) and decreases in resting and total metabolic rate (Fig. SI3e), including slight reductions in allometric exponents (Fig. SI3f). Model predicted allometric exponent for resting metabolic rate is not very far from previous experimental estimates for house crickets (0.899, [44]).

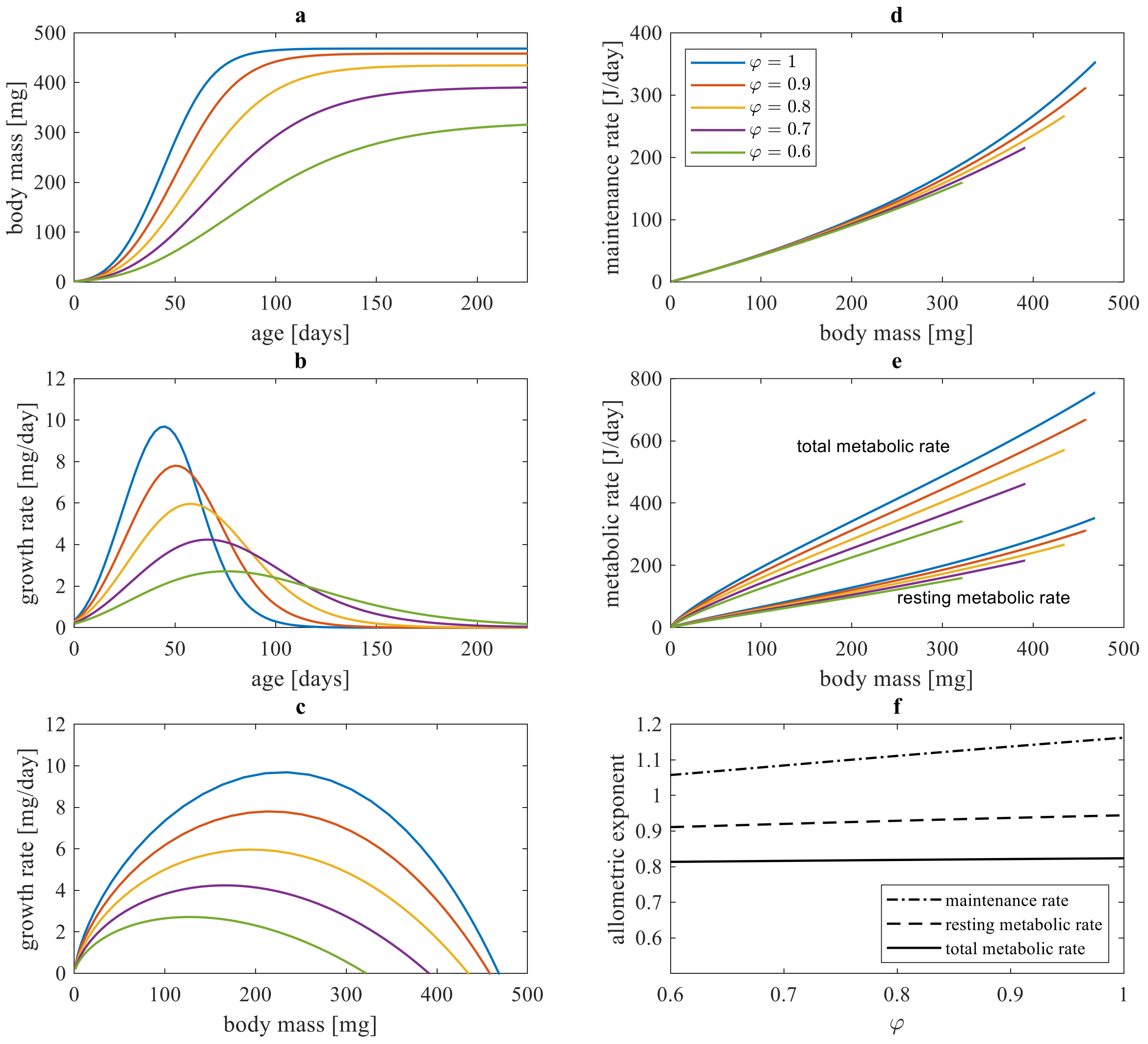

Fig. SI3: Predictions of MGM with varying fixed relative food acquirement φ (see legend in subplot **d**, other parameter values according to Table SI2).

MGM predictions of the age (t_{70}) when 70 % of the ultimate body mass is reached for varying relative food acquirement φ are shown in Fig. SI4. The pattern is highly dependent on the relative defence allocation exponent b_N, a model parameter that describes how fast relative allocation to negotiable maintenance costs increases with body mass. With $b_N = 0$, representing linear allometric scaling of maintenance costs (in similarity with common mechanistic growth models), t_{70} is roughly unchanged when φ is decreased below the ad libitum level ($\varphi = 1$). This is in line with the OGM for food restriction, according to which animals reach a specified fraction of the ultimate body mass at the same age regardless of the level of food limitation, a prediction that has been empirically verified for mammals [13]. With $b_N \gtrsim 0.5$ (considerable hyperallometric scaling of maintenance costs), t_{70} increases with decreased food availability. This is more in line with empirical data for house crickets (Fig. 3c).

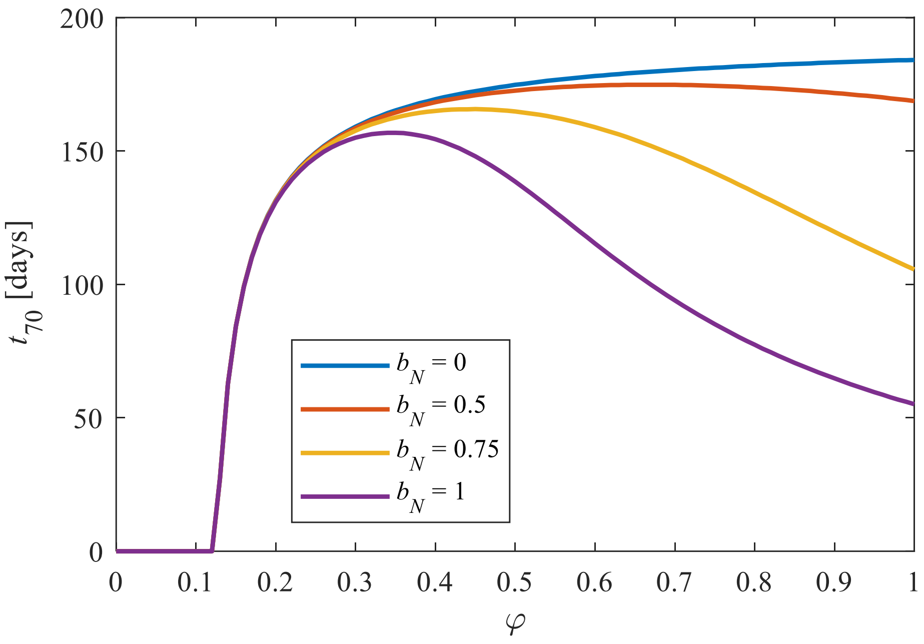

Fig. SI4: MGM predictions of t_{70} (the age when 70 % of the ultimate body mass is reached) vs. relative food acquirement φ for different values of the relative defence allocation exponent b_N.

2.3 EFFECTS OF FEEDING COSTS

As expected, with increased feeding costs, MGM predicts decreased ultimate body mass (Fig. SI5a) and decreased growth rates (Fig. SI5bc). The direct effect of increased feeding costs (Fig. SI5d) combined with reduced resting metabolic rate as a consequence of reduced growth overhead costs (Fig. SI5e), results in increased total metabolic rate with altered allometric scaling (Fig. SI5f).

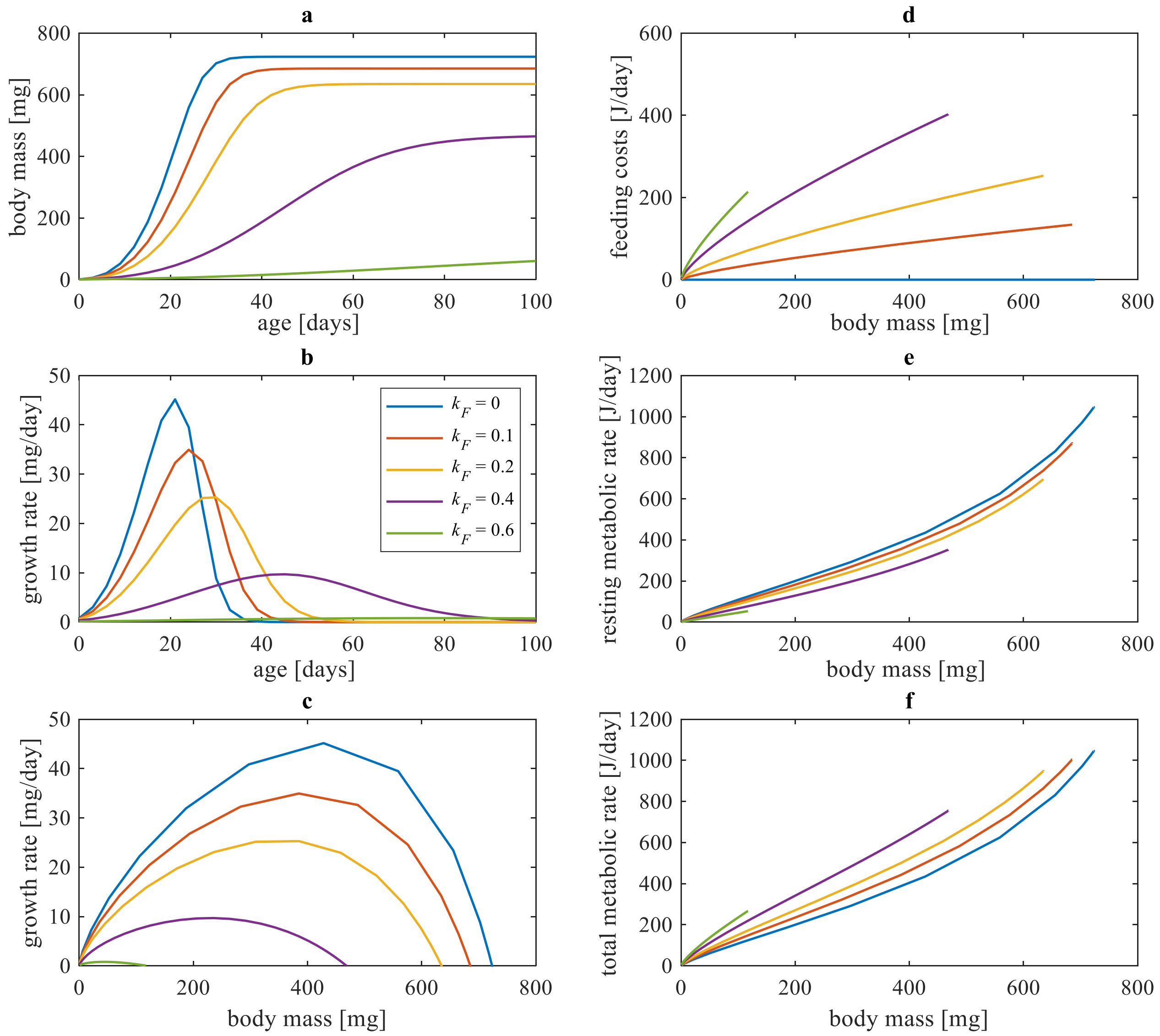

Fig. SI5: Predictions of MGM with varying relative feeding cost k_F (see legend in subplot **b**, other parameter values according to Table SI2).

2.4 EFFECTS OF BODY COMPOSITION

To demonstrate how body composition may affect predictions of MGM, the assumption of constant biomass energy density E_M was abandoned and replaced by the parameters in Table SI6. The switch model (Eqs. (SI17) and (SI18)) was applied, assuming that growth is either completely somatic or completely reproductive. The growth fractions f_L and f_{CP} were described by allometric relations (Eq. (SI20)), assuming equal growth exponents for carbohydrates/proteins and lipids ($b_{CP} = b_L = b_{CPL}$). The common growth exponent b_{CPL} describes how fast the fraction of lipid synthesis increases and how fast the fraction of carbohydrate/protein synthesis decreases with body mass. The biomass energy density was described by Eq. (SI23).

Table SI6: Additional MGM parameter values applied in numerical simulations (Fig. SI6) to demonstrate effects of body composition.

Parameter	Notation	Value	Unit
Carbohydrate/protein energy density	E_{CP}	17	J/mg
Lipid energy density	E_L	37	J/mg
Reproductive tissue energy density	E_{MR}	8.0	J/mg
Initial relative carbohydrate/protein growth	f_{CP0}	0.15	-
Relative lipid growth at maturation	f_{LM}	0.15	-

As a consequence of f_{LM} and f_{CP0} being fixed, the energy density of newly synthesized biomass E_M was fixed at birth and maturation. The variation of E_M in between does not affect ultimate body mass (Fig. SI6a), but growth rates are increased and peaks at a smaller body mass (Fig. SI6bc) when E_M is decreased as a consequence of increased b_{CPL} (Fig. SI6d), representing a more delayed growth of lipids (with twice the energy density of carbohydrates and proteins).

At maturation, when somatic growth is replaced by synthesis of reproductive tissue with higher energy density, growth rates are instantaneously decreased (Fig. SI6bc) as a consequence of the switch model that assumes instantaneously increased energy density of new biomass (Fig. SI6d). A successive onset of maturation (Eq. SI9-10) would instead result in a more gradual change in growth rate and energy density of newly synthesized biomass. Effects on allometries for resting and total metabolic rate are minor (Fig. SI6ef). Model predicted allometric exponent for resting metabolic rate is not very far from previous experimental estimates for house crickets (0.899, [44]).

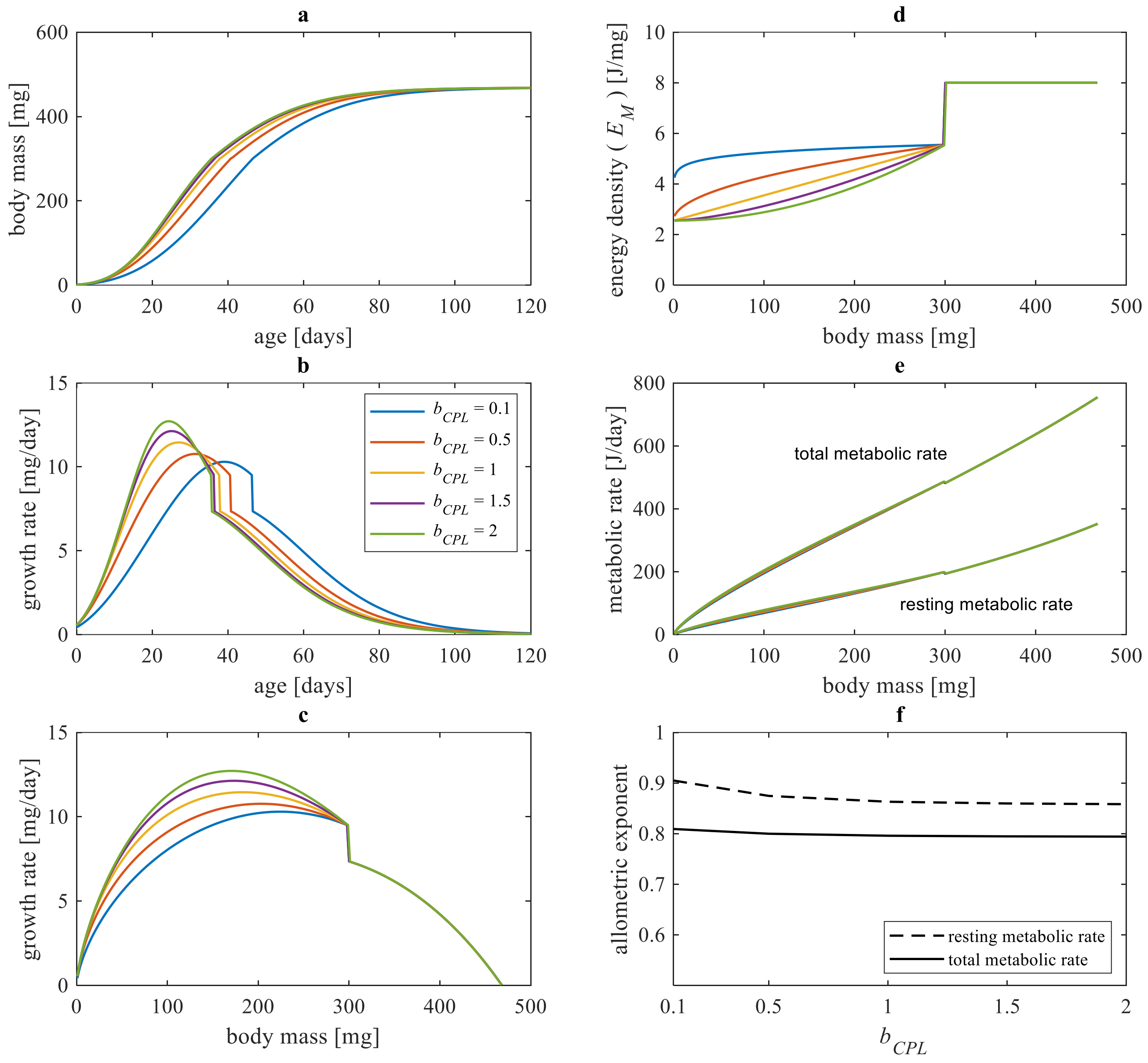

Fig. SI6: Predictions of MGM with varying growth exponent b_{CPL} (see legend in subplot **b**), but fixed fractions of relative growth at birth and maturation (f_{LM} and f_{CP0} fixed) under the switch model (Eqs. (SI17)-(SI18), assuming instantaneous switch from pure somatic to pure reproductive growth).

2.5 Effects of growth strategy

In MGM, type of cell growth (cell division or cell enlargement) may affect growth patterns in two ways; 1) through differentiated overhead costs for cell division and cell and enlargement and 2) through changes in average cell size, affecting relative contributions from surface-dependent and volume-dependent processes to total maintenance costs. To demonstrate how growth strategy may affect predictions of MGM, the assumption of constant specific growth overhead cost E_S and constant specific basal maintenance cost γ_B were abandoned and replaced by the parameters in Table SI7. The switch model (Eqs. (SI17) and (SI18), assuming instantaneous switch from pure somatic to pure reproductive growth) was applied. Relative somatic growth due to cell enlargement f_G was described by Eq. (SI22), specific growth overhead cost E_S by Eq. (SI26) and specific somatic basal maintenance cost γ_{BS} by Eq. (SI37). Furthermore, the surface- dependent cost for basal maintenance of somatic cells $\tilde{\gamma}_{BSA}$ was adjusted to obtain a fixed specific basal somatic maintenance cost at maturation γ_{BSM}.

Table SI7: Additional MGM parameter values applied in numerical simulations (Figs. SI7-SI8) to demonstrate effects of growth strategy.

Parameter	Notation	Value	Unit
Specific overhead cost for cell division of somatic tissue	E_{SSD}	2.0	J/mg
Specific overhead cost for cell enlargement of somatic tissue	E_{SSG}	4.0	J/mg
Specific overhead cost for growth of reproductive tissue	E_{SR}	4.0	J/mg
Relative somatic growth due to cell enlargement at maturation	f_{GM}	0.5	-
Initial cell mass	m_{C0}	$1\cdot10^{-6}$	mg
Volume-dependent specific somatic basal maintenance rate	γ_{BSV}	0.25	J/mg

With fixed proportions of cell enlargement and cell division (b_G = 0), $f_G = f_{GM}$ is the proportion of somatic growth that is due to cell enlargement. Corresponding predictions are shown in Fig. SI7. Effects of varying f_G for different b_G and different relative cost of volume-dependent processes are shown in Fig. SI8.

Ultimate body mass is independent of f_G since the specific somatic basal maintenance rate at maturation γ_{BSM} has been fixed (Fig. SI7a). With fixed proportions of cell enlargement and cell division throughout the growth period, increased relative contribution from cell enlargement results in slightly lowered growth rate peaking at somewhat larger body mass (Fig. SI7bc). Metabolic rates are barely affected (Fig. SI7def). Model predicted allometric exponent for resting metabolic rate is not very far from previous experimental estimates for house crickets (0.899, [44]).

As a consequence of decreased growth rate at fixed ultimate body mass, t_{95} increases with increased relative contribution from cell enlargement to somatic growth (f_G) if there are any costs to pay for surface-dependent processes ($f_{BSV} < 1$) (Fig. SI8). The pattern is a consequence of volume-dependent processes increasing faster with body mass than surface-dependent processes. With increasing proportion of somatic growth that is due to cell enlargement as body size increases ($b_G > 0$), t_{95} decreases with increasing b_G (Fig. SI8a). With large b_G most cell growth occurs close to maturation and cell division dominates early ages. The predicted pattern arises since surface-dependent costs increase slower with body mass compared to volume-dependent costs. Furthermore, t_{95} decreases with increasing relative cost of volume-dependent processes f_{BSV} (Fig. SI8b).

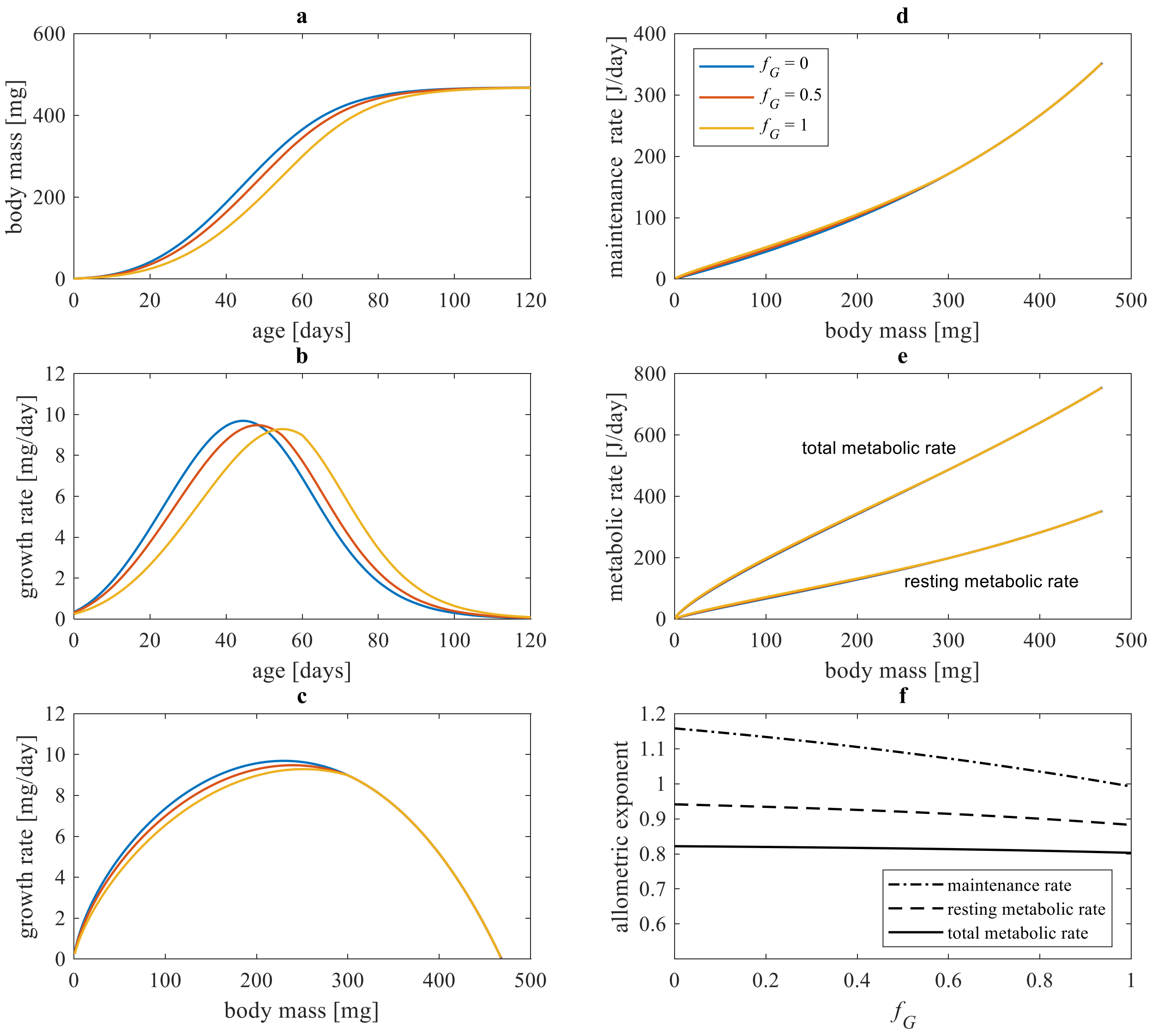

Fig. SI7: Predictions of MGM with varying fixed proportions of somatic growth due to cell enlargement f_G (see legend in subplot **d**) with $b_G = 0$ so that $f_G = f_{GM}$.

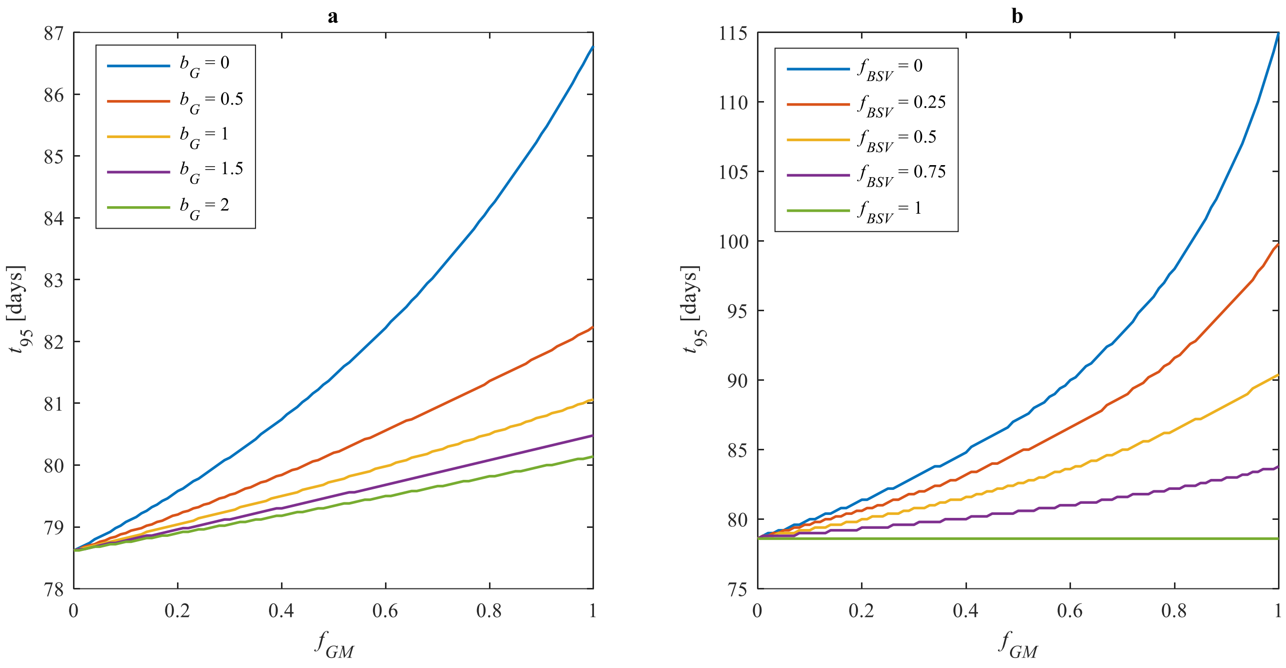

Fig. SI8: Predicted t_{95} with varying f_{GM} and different fixed values of **a**) b_G and **b**) f_{BSV}, where t_{95} is the age when 95 % of ultimate body mass is reached, f_{GM} is relative somatic growth due to cell enlargement at maturation, b_G is the cell growth exponent and $f_{BSV} = \gamma_{BSV}/\gamma_{BSM}$ is the relative cost of volume-dependent processes. With $b_G = 0$, the proportion of somatic growth that is due to cell enlargement is fixed ($f_G = f_{GM}$), while for $b_G > 0$, f_G increases with body mass towards f_{GM}.

2.6 Effects of allocation to negotiable maintenance

To illustrate how the strategy of allocation to negotiable maintenance costs may affect predictions of MGM, it was first assumed that a fixed proportion of total maintenance costs is allocated to negotiable maintenance costs at ultimate body mass, i.e. applying a fixed $\rho_N(W_U) = a_N W_U^{b_N}$ (Eq. (SI8)),which indirectly fixates ultimate body mass W_U (Fig. SI9a).

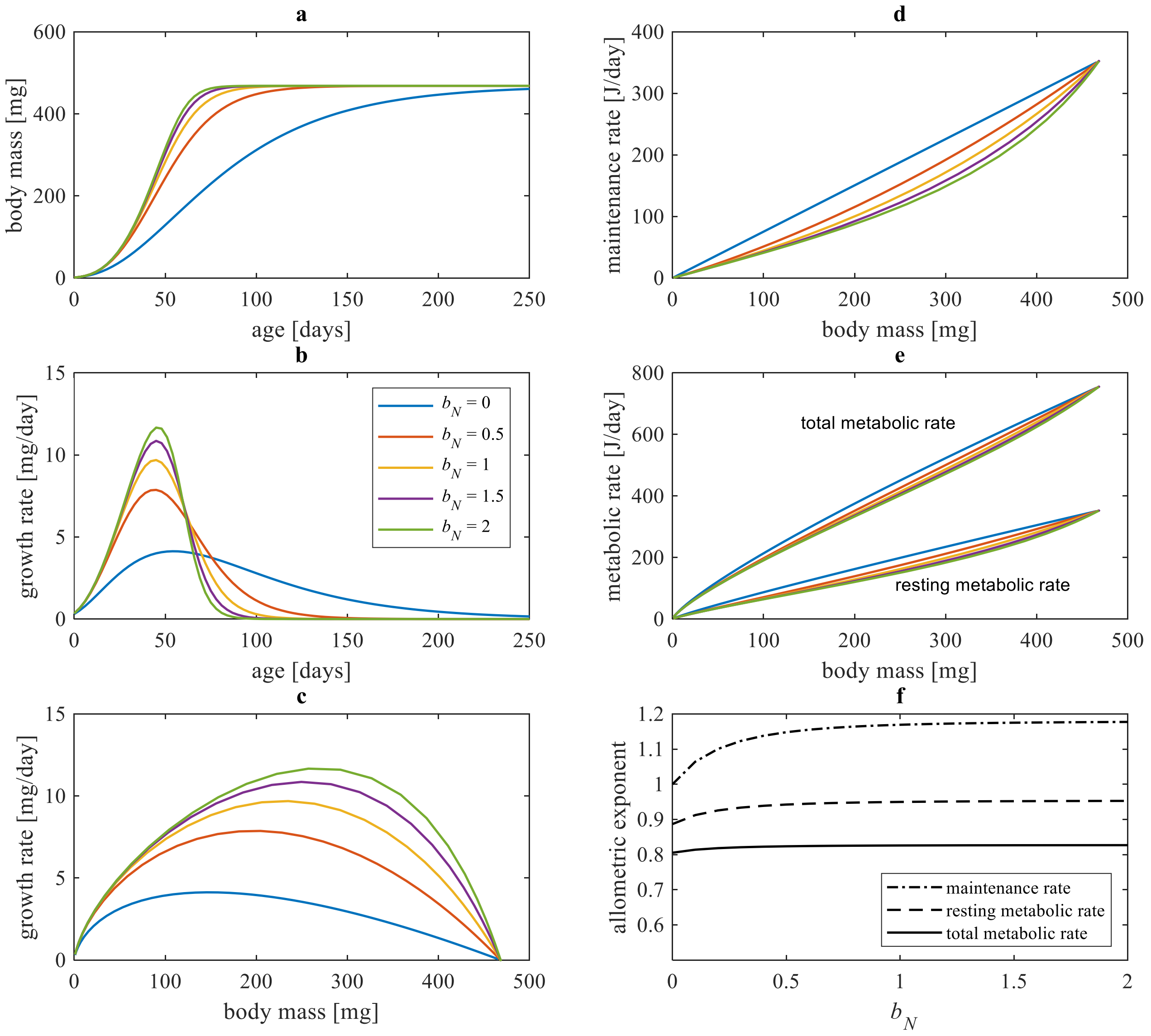

Fig. SI9: Predictions of MGM with fixed $\rho_N(W_U) = a_N W_U^{b_N}$ (a fixed proportion of total maintenance costs is allocated to negotiable maintenance costs at ultimate body mass), but varying relative defence allocation exponent b_N (see legend in subplot **b**, other parameter values according to Table SI2).

Increasing relative defence allocation during growth ($b_N > 0$) yields a pattern of initially fast growth and a large peak in growth rate followed by rapid decrease, as observed in house crickets (Fig. SI1b) but not captured by GSGM (corresponding to $b_N = 0$). With increased b_N, maximum growth rate increases and occurs at larger body mass (Fig. SI9bc). The relative body mass at maximum growth rate $\omega^* = W^*/W_U$ thus increases with increased b_N, a pattern that enabled accurate fit between MGM and empirical growth curve for house crickets [41] by applying $b_N = 1$ instead of $b_N = 0$ (corresponding to GSGM). The pattern occurs because large b_N results in maintenance costs that are initially low and increase fast with body mass at the end. Variation of b_N under fixed W_U has some (but not very large) effects on allometries for metabolic rates (Fig. SI9de). Maintenance rate (R_M) scales super-linearly with body mass (W) for $b_N > 0$ ($R_M \approx a_M W^{b_M}$ with $b_M > 1$), whereas resting metabolic rate (R_R) and total metabolic rate (R_{tot}) scale sub-linearly ($R_R \approx a_R W^{b_R}$, $b_R < 1$, $R_{tot} \approx a_T W^{b_T}$, $b_T < 1$) for all b_N (Fig. SI9f). Model predicted allometric exponent for resting metabolic rate is not very far from previous experimental estimates for house crickets (0.899, [44]).

MGM predictions of t_{95} versus W_{95} for varying a_N and b_N are shown in Fig. SI10. If b_N is increased (resulting in faster increase of relative defence allocation with body mass) for a fixed a_N, ultimate body mass W_U is decreased since maintenance costs successively turn larger. Also, if a_N is increased (resulting in increased fraction of total maintenance costs that are allocated to negotiable costs) for a fixed b_N, W_U is decreased. The age t_{95} required to reach $W_{95} = 0.95W_U$ is positively correlated with W_{95} (less time is required to reach a lower W_{95}).

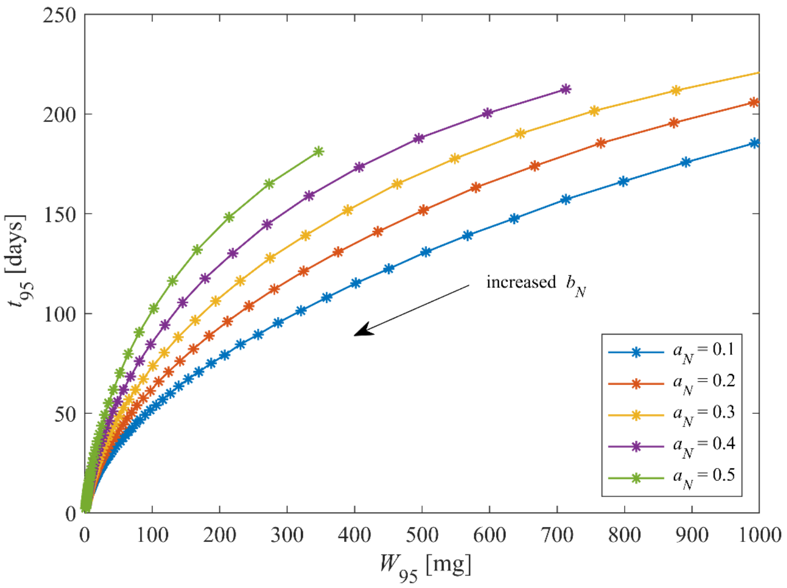

Fig. SI10 MGM predictions of t_{95} vs. W_{95} with different fixed values of a_N (relative defence normalisation constant) and b_N (relative defence allocation exponent). Here b_N is varied on an equidistant scale in the interval $0 \leq b_N \leq 1$ and each star ($*$) represents a value, with the right-most star in each curve corresponding to $b_N = 0$, and b_N successively increasing towards the left.

2.7 Effects of allocation to reproduction

To illustrate how increasing allocation to reproduction during growth affects predictions of MGM, it was assumed that growth of reproductive tissue (gonads, sperms, eggs, reproductive buffer) constitute an increasing fraction f_R of total growth (Eq. (SI14)), described by an allometric relation between birth and ultimate size, $f_R = \eta_R W^{b_R}$ (Eq. (SI15) with $W_{mat} = 0$), with growth finally completely reproductive ($f_{RU} = 1$). Maturation mass was put to zero in order to describe allocation to reproduction by an allometry starting at birth. In addition, it was assumed that reproductive tissue is twice as expensive to synthesize as somatic tissue ($E_{MR} = 2E_{MS}$, $E_{SR} = 2E_{SS}$, Table SI8). The energy density E_M and the specific growth overhead cost E_S for all tissue (somatic and reproductive) were calculated with Eqs. (SI23) and (SI26).

Table SI8: Additional MGM parameter values applied in numerical simulations (Fig. SI11) to demonstrate effects of allocation to reproduction.

**Parameter**	**Notation**	**Value**	**Unit**
Maturation mass	W_{mat}	0	mg
Energy density of somatic tissue	E_{MS}	6.0	J/mg
Energy density of reproductive tissue	E_{MR}	12.0	J/mg
Specific overhead cost for growth of somatic tissue	E_{SS}	3.0	J/mg
Specific overhead cost for growth of reproductive tissue	E_{SR}	6.0	J/mg
Ultimate reproductive growth fraction	f_{RU}	1	-

The relative reproductive growth normalisation constant η_R (Eq. (SI15)) was adjusted to keep ultimate body mass fixed under varying allometric exponent b_R. With increased b_R, total growth (somatic + reproductive) occurs faster, peaking at a higher maximum growth rate (Fig. SI11abc). Age and body mass at maximum growth rate are also affected by b_R (Fig. SI11bc). When relative reproductive growth is proportional to body mass ($b_R = 1$), total specific growth costs ($E_M + E_S$) increase linearly with body mass (Fig. SI11d). With hyperallometric relative reproductive growth ($b_R > 1$), $E_M + E_S$ increase faster than linearly with body mass (Fig. SI11d) and the total reproductive cost is initially hyperallometric (Fig. SI11e). With hypoallometric relative reproductive growth ($b_R < 1$), these patterns are reversed (Fig. SI11de). With increased b_R, the total reproductive cost is decreased and skewed towards the end (Fig. SI11e). Allometric scaling of resting and total metabolic rates are not affected (Fig. SI11f).

In order to investigate if hyperallometric relative reproductive growth ($b_R > 0$), instead of hyperallometric allocation to negotiable maintenance ($b_N > 0$), can explain observed growth curves in house crickets reared at near ad libitum, Eq. (SI8) with $b_N = 0$ (and $\varphi = 1$) was combined with E_M and E_S from Eqs. (SI23) and (SI26), and $f_R = \eta_R W^{b_R}$, yielding the following growth equation:

$$\frac{dW}{dt}=\frac{aW^b-cW}{1+\tilde{\eta}_R W^{b_R}} \quad , \quad \begin{cases} a=\frac{e\alpha-k_F}{E_{MS}+E_{SS}} \quad , \quad b=\beta \quad , \quad c=\frac{\gamma_B/(1-a_N)}{E_{MS}+E_{SS}} \\ \tilde{\eta}_R=\frac{E_{MR}+E_{SR}-(E_{MS}+E_{SS})}{E_{MS}+E_{SS}}\eta_R \end{cases} \tag{SI13}$$

Application of a, b, c, $\tilde{\eta}_R$ and b_R as free parameters in the optimization procedure used for fitting models to data for house crickets at near ad libitum [41], resulted in $\tilde{\eta}_R \approx 0$ and same values for a, b and c as obtained for GSGM (Table SI3). The best fit to data is thus equivalent to GSGM and hyperallometric allocation to reproduction does nothing to improve it.

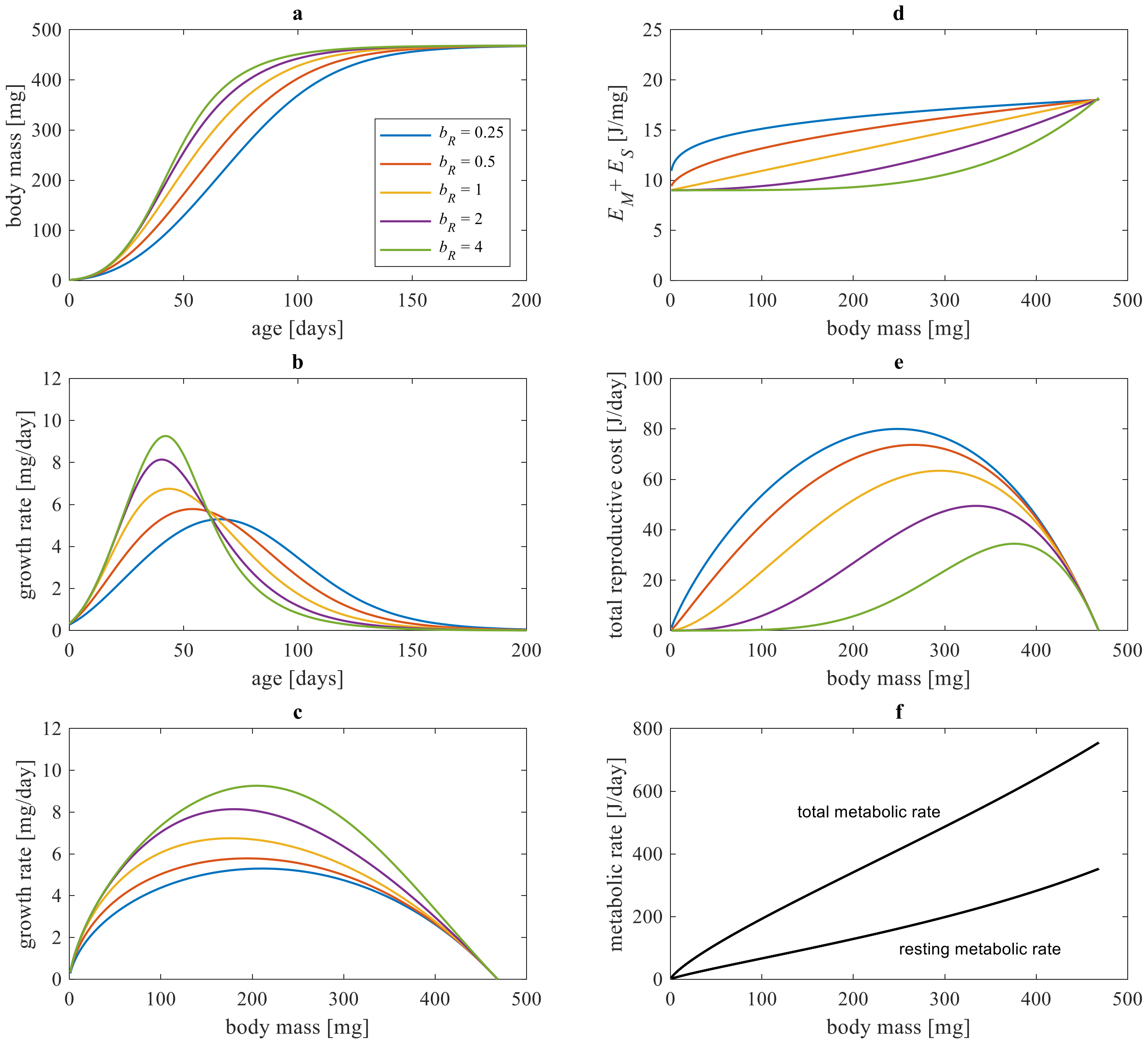

Fig. SI11: Predictions of MGM with fixed ultimate body mass and varying allometric exponent for relative reproductive growth b_R (see legend in subplot **a**).

3. Inclusion of additional effects in developed growth model

Main section 5 (Methods) derives the main components of MGM and suggests basic alternatives for each component that leads to the simplest relevant full version of MGM (Eq. 30). Below, we suggest how more complex and/or additional features, accounting for (i) reproductive growth, (ii) changes in somatic body composition, (iii) somatic growth strategy and (iv) variability in growth overhead costs and basal somatic maintenance costs, could be included in MGM if considered relevant.

3.1 Reproductive growth

If reproductive tissue has different energy density and/or overhead costs, it may be relevant to account for reproductive growth during ontogeny. This can be done in numerous ways, but we here outline one possible approach. The growth of reproductive tissue may be described as a mass-dependent fraction $f_R(W)$ of total body growth:

$$\frac{dW_R}{dt} = f_R(W) \cdot \frac{dW}{dt} \tag{SI14}$$

Reproductive growth may start at a certain life stage, such as maturation. The reproductive growth fraction f_R may then be described by an allometric function:

$$f_R = \begin{cases} 0 & , \quad W < W_{mat} \\ \eta_R (W - W_{mat})^{b_R} & , \quad W \geq W_{mat} \end{cases} \quad , \quad \eta_R = \frac{f_{RU}}{(W_U - W_{mat})^{b_R}} \qquad (0 \leq f_{RU} \leq 1 \, , b_R \geq 0) \tag{SI15}$$

Here, W_{mat} is the maturation mass and W_U is the ultimate body mass. The ultimate reproductive growth fraction f_{RU} and the allometric exponent b_R are constants. Integration of Eq. (SI14) yields the reproductive body mass W_R and the proportion of reproductive tissue ($p_R = W_R/W$) is obtained as:

$$p_R = \begin{cases} 0 & , \quad W < W_{mat} \\ \dfrac{\eta_R}{(b_R + 1)W} (W - W_{mat})^{b_R} & , \quad W \geq W_{mat} \end{cases} \tag{SI16}$$

Eqs. (SI15) and (SI16) can then be inserted into expressions for energy content (Eq. (13) or (SI23)), growth overhead costs (Eq. (14) or (SI26)) and maintenance (Eqs. (21) and (22)).

Eqs. (SI15)-(SI16) describe a maturation process where somatic growth is gradually replaced by reproductive growth. However, many mathematical complications can be avoided under the *switch model*, where pure somatic growth is replaced by pure reproductive growth at some point $W = W_{mat}$:

$$f_R = \begin{cases} 0 & , \quad W < W_{mat} \\ 1 & , \quad W \geq W_{mat} \end{cases} \tag{SI17}$$

The proportion p_R of reproductive tissue is then:

$$p_R = \begin{cases} 0 & , \quad W < W_{mat} \\ 1 - W_{mat}/W & , \quad W \geq W_{mat} \end{cases} \tag{SI18}$$

3.2 Growth of somatic tissue components

If the relative proportions of lipids, carbohydrates and proteins in somatic tissue changes significantly during ontogeny it may be relevant to account for this. Growth of somatic tissue can be divided into growth of lipids and growth of carbohydrates and proteins (with similar energy density). The growth rates can be described as mass-dependent fractions f_L and f_{CP} of the total somatic growth rate:

$$\frac{dW_{SL}}{dt} = f_L(W_S) \cdot \frac{dW_S}{dt} \quad , \quad \frac{dW_{SCP}}{dt} = f_{CP}(W_S) \cdot \frac{dW_S}{dt} \tag{SI19}$$

The growth fractions f_L and f_{CP} may be described by allometric functions. Generally, animals increase their proportion of lipid-rich reserve during growth [11]. By assuming that somatic lipid synthesis is initially zero (at zero body mass) and increases with somatic growth, and that somatic carbohydrate/protein synthesis is zero at maturation, the allometries can be expressed as:

$$\begin{aligned} & f_L(W_S) = \eta_L W_S^{b_L} \quad , \quad \eta_L = f_{LM} / W_{SM}^{b_L} \qquad (0 \leq f_{LM} \leq 1 \ , \ b_L \geq 0) \\ & f_{CP}(W_S) = \begin{cases} f_{CP0} & , \quad b_{CP} = 0 \\ f_{CP0} - \eta_{CP} W_S^{b_{CP}} & , \quad b_{CP} > 0 \end{cases} \quad , \quad \eta_{CP} = f_{CP0} / W_{SM}^{b_{CP}} \quad , \quad (0 \leq f_{CP0} \leq 1) \end{aligned} \tag{SI20}$$

Here W_{SM} is the somatic body mass at maturation. The lipid growth fraction at maturation f_{LM}, the initial carbohydrate/protein growth fraction f_{CP0} and the allometric exponents (b_L, b_{CP}) are constants. Eq. (SI20) can then be inserted into expressions for energy content (Eq. (13) or (SI23)).

3.3 Somatic growth due to cell enlargement and cell division

If somatic growth results from a combination of cell growth and cell division that changes significantly during ontogeny this may need to be accounted for. Somatic growth due to cell enlargement can be described as a mass-dependent fraction f_G of total somatic growth:

$$\frac{dW_{SG}}{dt} = f_G(W_S) \cdot \frac{dW_S}{dt} \tag{SI21}$$

Generally, animals increase body mass mainly through cell division during early ontogeny and mainly through cell enlargement at later life stages [45]. If cell enlargement constitutes an increasing fraction of total somatic growth with increased body mass (with a corresponding decreasing relative contribution of cell division), f_G may be expressed as an allometric function of somatic body mass:

$$f_G = \eta_G W_S^{b_G} \quad , \quad \eta_G = f_{GM} / W_{SM}^{b_G} \qquad (0 \le f_{GM} \le 1 \ , \ b_G \ge 0) \qquad \text{(SI22)}$$

The somatic cell growth fraction at maturation f_{GM} and the allometric exponent b_G are constants. Eq. (SI22) can then be inserted into expressions for growth overhead costs (Eq. (14) or (SI26)).

3.4 ENERGY BOUNDED IN SYNTHESIZED BIOMASS

The rate at which energy is bounded in synthesized biomass (G) is proportional to the growth rate (dW/dt). It is composed of energy bounded in synthesized somatic tissue (with energy density E_{MS}) and energy bounded in synthesized reproductive tissue (with energy density E_{MR}). The somatic tissue is divided into carbohydrates/proteins (with energy density $E_{CP} \approx 17$ J/mg [46]), lipids (with energy density $E_L \approx 37$ J/mg [46]) and others (with zero energy density):

$$G = E_M(W) \cdot \frac{dW}{dt} \quad , \quad E_M(W) = E_{MS}(1 - f_R) + E_{MR} f_R = (E_{CP} f_{CP} + E_L f_L)(1 - f_R) + E_{MR} f_R \qquad \text{(SI23)}$$

By applying the switch model (Eq. (SI17)) and the allometries in Eq. (SI20), the biomass energy density is obtained as:

$$E_M(W) = \begin{cases} E_{CP}(f_{CP0} - \eta_{CP} W^{b_{CP}}) + E_L \eta_L W^{b_L} & , \ W < W_{mat} \\ E_{MR} & , \ W \ge W_{mat} \end{cases} \qquad \text{(SI24)}$$

Since the relative amount of lipids in somatic tissue increases during growth, the biomass energy density increases during ontogeny.

The average energy density of new synthesized body tissue (E_M) should not be confused with the average energy density ($\bar{E}_M$) of biomass that has already been synthesized. With constant body composition, the two are equal and constant ($E_M = \bar{E}_M$), but in a general case they are related as:

$$E_M(W) = \frac{d}{dW}(\bar{E}_M \cdot W) \quad \Leftrightarrow \quad \bar{E}_M = \frac{1}{W} \int_{W_0}^{W} E_M(W) dW \qquad \text{(SI25)}$$

Here W_0 is the birth mass.

3.5 Growth overhead costs

Growth overhead costs (R_G) include all indirect costs of growth (such as assembling macromolecules from monomers) and are proportional to the growth rate (dW/dt). They are divided into costs for producing somatic tissue (E_{SS}) and costs for producing reproductive tissue (E_{SR}). Overhead costs for somatic growth are further divided into costs for cell division (E_{SSD}) and cell enlargement (E_{SSG}):

$$R_G = E_S(W)\cdot\frac{dW}{dt} \quad , \quad E_S(W) = E_{SS}(1-f_R) + E_{SR}f_R = \left[E_{SSD}(1-f_G) + E_{SSG}f_G\right](1-f_R) + E_{SR}f_R \quad \text{(SI26)}$$

By applying the switch model (Eq. (SI17)) and the allometry in Eq. (SI22), the specific growth overhead cost is obtained as:

$$E_S(W) = \begin{cases} E_{SSD}(1-\eta_G W^{b_G}) + E_{SSG}\eta_G W^{b_G} & , \quad W < W_{mat} \\ E_{SR}W^{b_R} & , \quad W \geq W_{mat} \end{cases} \quad \text{(SI27)}$$

Since the relative contribution from cell enlargement to somatic growth increases as the organism grows, overhead costs for somatic cell growth becomes increasingly important during ontogeny.

3.6 Basal somatic maintenance cost

Maintenance of somatic tissue may be one of the more complex and variable parts of ontogenetic growth, being affected by a combination of internal and external factors and differing among organisms due to life history strategy. Accounting for this in a general way is difficult, but we here outline one suggestion for how the basal (non-negotiable) maintenance of somatic tissue is affected by growth strategy due to changes in average cell volume. Many maintenance processes (and costs related to them) can be expected to be proportional to cell volume (such as oxidative processes, protein synthesis and glycogenesis). However, a significant amount of basal maintenance are processes where costs can be expected to be proportional to cell surface area, such as maintenance of ion potentials across membranes [47]. If the organism grows by a combination of cell division and cell enlargement, the average cell size may change as the organism grows and thus also the mass-specific basal maintenance costs. The basal maintenance costs for somatic tissue R_{MBS} is here divided into a part proportional to average cell volume and a part proportional to average cell surface area, where cell volume is proportional to cell mass (m_C) and cell surface area is proportional to $m_C^{2/3}$:

$$R_{MBS} = N_C(\gamma_{BSV}\cdot m_C + \tilde{\gamma}_{BSA}\cdot m_C^{2/3}) \quad \text{(SI28)}$$

Here N_C is the total number of somatic cells. The total somatic body mass is the total number of somatic cells (N_C) multiplied by the average cell mass (m_C):

$$W_S = N_C m_C \tag{SI29}$$

Differentiation with respect to time yields one part that is due to cell enlargement and one part that is due to cell division:

$$\frac{dW_S}{dt} = \frac{dW_{SG}}{dt} + \frac{dW_{SD}}{dt} \quad , \quad \begin{cases} \frac{dW_{SG}}{dt} = N_C \frac{dm_C}{dt} \\ \frac{dW_{SD}}{dt} = m_C \frac{dN_C}{dt} \end{cases} \tag{SI30}$$

By describing somatic growth due to cell enlargement as an allometric fraction of total somatic growth (Eqs. (SI21)-(SI22)), somatic growth due to cell enlargement and cell division are obtained as:

$$\begin{cases} \frac{dW_{SG}}{dt} = \eta_G W_S^{b_G} \frac{dW_S}{dt} \\ \frac{dW_{SD}}{dt} = (1 - \eta_G W_S^{b_G}) \frac{dW_S}{dt} \end{cases} \quad , \quad \eta_G = f_{GM} / W_{SM}^{b_G} \tag{SI31}$$

Comparing the expressions for somatic growth due to cell enlargement (dW_{SG}/dt) between Eqs. (SI30) and (SI31) yields:

$$N_C \frac{dm_C}{dt} = \eta_G W_S^{b_G} \frac{dW_S}{dt} \tag{SI32}$$

Integration (with $N_C = W_S / m_C$) yields:

$$\int_{m_{C0}}^{m_C} \frac{dm_C}{m_C} = \eta_G \int_{m_{C0}}^{W_S} W_S^{b_G - 1} dW_S \tag{SI33}$$

The lower integration limit corresponds to the initial state of a single somatic cell with mass m_{C0}. An approximation for the average somatic cell mass is obtained by assuming that the initial cell mass is small compared to the total somatic mass W_S ($m_{C0} \approx 0$):

$$m_C \approx \begin{cases} m_{C0} (W_S / m_{C0})^{f_{GM}} & , \quad b_G = 0 \\ m_{C0} \cdot \exp\left[\frac{f_{GM}}{b_G} \left(\frac{W_S}{W_{mat}} \right)^{b_G} \right] & , \quad b_G > 0 \end{cases} \tag{SI34}$$

Insertion of Eq. (SI34) into Eq. (SI28) with $N_C = W_S / m_C$ yields the total basal somatic maintenance cost as:

$$R_{MBS} \approx \begin{cases} \gamma_{BSV} \cdot W_S + \gamma_{BSA} \cdot W_S^{1-f_{GM}/3} & , \quad b_G = 0 \\ \gamma_{BSV} \cdot W_S + \gamma_{BSA} \cdot W_S \cdot \exp\left[-\frac{f_{GM}}{3b_G} \cdot \left(\frac{W_S}{W_{mat}} \right)^{b_G} \right] & , \quad b_G > 0 \end{cases} \qquad \text{(SI35)}$$

The volume-dependent and area-dependent specific somatic basal maintenance costs (γ_{BSV} and γ_{BSA}) are constants.

$$\gamma_{BSA} = \begin{cases} \tilde{\gamma}_{BSA} / m_{C0}^{(1-f_{GM})/3} & , \quad b_G = 0 \\ \tilde{\gamma}_{BSA} / m_{C0}^{1/3} & , \quad b_G > 0 \end{cases} \qquad \text{(SI36)}$$

By applying the switch model (Eq. (SI18)), the specific somatic basal maintenance cost is obtained as:

$$\gamma_{BS}(W) \approx \begin{cases} \gamma_{BSV} + \gamma_{BSA} \cdot W^{-f_{GM}/3} & , \quad b_G = 0 \\ \gamma_{BSV} + \gamma_{BSA} \cdot \exp\left[-\frac{f_{GM}}{3b_G} \cdot \left(\frac{W}{W_{mat}} \right)^{b_G} \right] & , \quad b_G > 0 \end{cases} \qquad (W < W_{mat}) \qquad \text{(SI37)}$$

Since the average cell size increases as the organism grows, surface-dependent costs decrease in relation to volume-dependent costs during ontogeny, resulting in a decreasing specific somatic basal maintenance cost γ_{BS} with increased body mass.

References

1. Martyushev, L.M. & Terentiev, P.S. A universal model of ontogenetic growth. *Sci. Nat.* **102**, 29 (2015).
2. Schmalhausen, I. Beiträge zur quantitativen analyse der formbildung. II. Das problem des proportionalen wachstums. *Wilhelm Roux Arch Entwickl Mech Org* **110**, 33–62 (1927).
3. Verhulst, P.-F. Notice sur la loi que la population poursuit dans son accroissement. *Correspondance mathématique et physique* **10**, 113–121 (1838).
4. Gompertz, B. On the nature of the function expressive of the law of humanmortality, and on a new mode of determining the value of life contingencies. *Phil Trans R Soc* **115**, 513–585 (1825).
5. Pütter, A. Studien über physiologische Ähnlichkeit. VI. Wachstumsähnlichkeiten. *Pflügers Archiv für die gesamte Physiologie des Menschen und der Tiere* **180**, 298–340 (1920).
6. von Bertalanffy, L. Quantitative laws in metabolism and growth. *Q Rev Biol* **32**, 217–231 (1957).
7. Pauly, D. Gill size and temperature as governing factors in fish growth: a generalization of von Bertalanffy's growth formula. (Institut für Meereskunde PY, Kiel, 1979).
8. Kooijman, S.A.L.M. Population dynamics on basis of budgets. The Dynamics of Physiologically Structured Populations. in *Lecture Notes in Biomathematics,* Vol. 68 (eds. Metz, J. & Diekmann, O.) 266-297 (Springer-Verlag, Berlin, 1986).
9. Nisbet, R.M., Muller, E.B., Lika, K. & Kooijman, S. From molecules to ecosystems through dynamic energy budget models. *J. Anim. Ecol.* **69**, 913-926 (2000).
10. Sousa, T., Domingos, T. & Kooijman, S. From empirical patterns to theory: a formal metabolic theory of life. *Philos. Trans. R. Soc. B-Biol. Sci.* **363**, 2453-2464 (2008).
11. Kearney, M.R. & White, C.R. Testing Metabolic Theories. *Am. Nat.* **180**, 546-565 (2012).
12. Kooijman, S.A.L.M. Quantitative aspects of metabolic organization: a discussion of concepts. *Philos. Trans. R. Soc. Lond. Ser. B-Biol. Sci.* **356**, 331-349 (2001).
13. Hou, C., Bolt, K.M. & Bergman, A. A general model for ontogenetic growth under food restriction. *Proc. R. Soc. B-Biol. Sci.* **278**, 2881-2890 (2011).
14. Hou, C., *et al.* Energy Uptake and Allocation During Ontogeny. *Science* **322**, 736-739 (2008).
15. West, G.B., Brown, J.H. & Enquist, B.J. A general model for ontogenetic growth. *Nature* **413**, 628-631 (2001).
16. West, G.B., Brown, J.H. & Enquist, B.J. Growth models based on first principles or phenomenology? *Funct. Ecol.* **18**, 188-196 (2004).
17. Gillooly, J.F., Charnov, E.L., West, G.B., Savage, V.M. & Brown, J.H. Effects of size and temperature on developmental time. *Nature* **417**, 70-73 (2002).
18. Sibly, R.M. & Brown, J.H. Toward a physiological explanation of juvenile growth curves. *J. Zool.* **311**, 286-290 (2020).
19. Burton, T., Killen, S.S., Armstrong, J.D. & Metcalfe, N.B. What causes intraspecific variation in resting metabolic rate and what are its ecological consequences? *Proc. R. Soc. B-Biol. Sci.* **278**, 3465-3473 (2011).
20. Makarieva, A.M., Gorshkov, V.G., Li, B.L. & Chown, S.L. Size- and temperature-independence of minimum life-supporting metabolic rates. *Funct. Ecol.* **20**, 83-96 (2006).

21. Roark, A.M. & Bjorndal, K.A. Metabolic rate depression is induced by caloric restriction and correlates with rate of development and lifespan in a parthenogenetic insect. *Exp. Gerontol.* **44**, 413-419 (2009).
22. O'Connor, K.I., Taylor, A.C. & Metcalfe, N.B. The stability of standard metabolic rate during a period of food deprivation in juvenile Atlantic salmon. *J. Fish Biol.* **57**, 41-51 (2000).
23. Ronning, B., *et al.* Food restriction in young Japanese quails: effects on growth, metabolism, plasma thyroid hormones and mRNA species in the thyroid hormone signalling pathway. *J. Exp. Biol.* **212**, 3060-3067 (2009).
24. Ramsey, J.J., Harper, M.E. & Weindruch, R. Restriction of energy intake, energy expenditure, and aging. *Free Radic. Biol. Med.* **29**, 946-968 (2000).
25. Hou, C., Bolt, K. & Bergman, A. A General Life History Theory for Effects of Caloric Restriction on Health Maintenance. *BMC Syst. Biol.* **5**, 13 (2011).
26. Jiao, L.H., *et al.* Food restriction alters energy allocation strategy during growth in tobacco hornworms (Manduca sexta larvae). *Sci. Nat.* **102**, 40 (2015).
27. Moe, B., Stolevik, E. & Bech, C. Ducklings exhibit substantial energy-saving mechanisms as a response to short-term food shortage. *Physiol. Biochem. Zool.* **78**, 90-104 (2005).
28. Brzek, P. & Konarzewski, M. Effect of food shortage on the physiology and competitive abilities of sand martin (Riparia riparia) nestlings. *J. Exp. Biol.* **204**, 3065-3074 (2001).
29. Moe, B., Brunvoll, S., Mork, D., Brobakk, T.E. & Bech, C. Developmental plasticity of physiology and morphology in diet-restricted European shag nestlings (Phalacrocorax aristotelis). *J. Exp. Biol.* **207**, 4067-4076 (2004).
30. Zhang, D.W., Xiao, Z.J., Zeng, B.P., Li, K. & Tang, Y.L. Insect Behavior and Physiological Adaptation Mechanisms Under Starvation Stress. *Front. Physiol.* **10**, 8 (2019).
31. Masoro, E.J. Dietary restriction and ageing. *J. Am. Geriatr. Soc.* **41**, 994-999 (1993).
32. Yu, B.P. How diet influences the aging process of the rat. *Proc. Soc. Exp. Biol. Med.* **205**, 97-105 (1994).
33. Hou, C. Increasing Energetic Cost of Biosynthesis during Growth Makes Refeeding Deleterious. *Am. Nat.* **184**, 233-247 (2014).
34. Mangel, M. & Stamps, J. Trade-offs between growth and mortality and the maintenance of individual variation in growth. *Evol. Ecol. Res.* **3**, 583-593 (2001).
35. De Block, M. & Stoks, R. Short-term larval food stress and associated compensatory growth reduce adult immune function in a damselfly. *Ecol. Entomol.* **33**, 796-801 (2008).
36. Morgan, I.J. & Metcalfe, N.B. Deferred costs of compensatory growth after autumnal food shortage in juvenile salmon. *Proc. R. Soc. B-Biol. Sci.* **268**, 295-301 (2001).
37. Metcalfe, N.B. & Monaghan, P. Compensation for a bad start: grow now, pay later? *Trends Ecol. Evol.* **16**, 254-260 (2001).
38. Dmitriew, C.M. The evolution of growth trajectories: what limits growth rate? *Biol. Rev.* **86**, 97-116 (2011).
39. Kearney, M.R. What is the status of metabolic theory one century after Pütter invented the von Bertalanffy growth curve? *Biol. Rev.* **96**, 557-575 (2020).
40. Makarieva, A.M., Gorshkov, V.G. & Li, B.L. Ontogenetic growth: models and theory. *Ecol. Model.* **176**, 15-26 (2004).
41. Jonsson, T. Metabolic theory predicts animal self-thinning. *J. Anim. Ecol.* **86**, 645-653 (2017).
42. Woodring, J., Clifford, C.W. & Beckman, B.R. Food utilization and metabolic efficiency in larval and adult house crickets. *Journal of Insect Physiology* **25**, 903-912 (1979).

43. Woodring, J., Roe, R.M. & Clifford, C.W. Relation of feeding, growth, and metabolism to age in the larval, female house cricket. *Journal of Insect Physiology* **23**, 207-212 (1977).
44. Krüger, F. Größenabhängigkeit des Sauerstoffverbrauches einheimischer Grillen. *Biologisches Zentralblatt* **77**, 581–588 (1958).
45. Verberk, W., *et al.* Shrinking body sizes in response to warming: explanations for the temperature-size rule with special emphasis on the role of oxygen. *Biol. Rev.* **96**, 247-268 (2021).
46. Peters, R.H. *The ecological implications of body size* (Cambridge University Press, 1983).
47. Clarke, A. & Fraser, K.P.P. Why does metabolism scale with temperature? *Funct. Ecol.* **18**, 243-251 (2004).
